# Clinical value of serum biomarkers, squamous cell carcinoma antigen and apolipoprotein C-II in follow-up of patients with locally advanced cervical squamous cell carcinoma treated with radiation: A multicenter prospective cohort study

**DOI:** 10.1371/journal.pone.0259235

**Published:** 2021-11-02

**Authors:** Yoko Harima, Takuro Ariga, Yuko Kaneyasu, Hitoshi Ikushima, Sunao Tokumaru, Shigetoshi Shimamoto, Takeo Takahashi, Noriko Ii, Kayoko Tsujino, Anneyuko I. Saito, Hiroki Ushijima, Takafumi Toita, Tatsuya Ohno

**Affiliations:** 1 Department of Radiology, Medical Center, Kansai Medical University, Osaka, Japan; 2 Department of Radiology, Graduate School of Medical Science, University of the Ryukyus, Okinawa, Japan; 3 Health Information Management Center, University of the Ryukyus Hospital, Okinawa, Japan; 4 Department of Radiation Oncology, Graduate School of Biomedical Sciences, Hiroshima University, Hiroshima, Japan; 5 Department of Radiation Oncology, National Hospital Organization Fukuyama Medical Center, Hiroshima, Japan; 6 Department of Therapeutic Radiology, Tokushima University Graduate School, Tokushima, Japan; 7 Department of Radiology, Hyogo Ion Beam Medical Center, Tatsuno, Japan; 8 Department of Radiation Oncology, Osaka General Medical Center, Osaka, Japan; 9 Department of Radiation Oncology, Saitama Medical Center, Saitama Medical University, Saitama, Japan; 10 Department of Radiation Oncology, Ise Red Cross Hospital, Mie Japan; 11 Department of Radiation Oncology, Hyogo Cancer Center, Hyogo, Japan; 12 Department of Radiation Oncology, Juntendo University Faculty of Medicine, Tokyo, Japan; 13 Department of Radiation Oncology, Saitama Cancer Center, Saitama, Japan; 14 Radiation Therapy Center, Okinawa Chubu hospital, Okinawa, Japan; 15 Department of Radiation Oncology, Gunma University Graduate School of Medicine, Gunma, Japan; Washington University in Saint Louis, UNITED STATES

## Abstract

There are currently no reliable, established serum biomarkers to predict the prognosis of radiotherapy for advanced cervical cancer. We aimed to identify serum biomarkers for survival after radiotherapy for cervical cancer. In this multicenter prospective cohort study, the usefulness of pre- and posttreatment serum protein levels of potential biomarkers, including squamous cell carcinoma antigen (SCC-Ag), apolipoprotein C-II (ApoC-II), matrix metalloproteinase (MMP)1, and MMP2, were evaluated together with clinical factors in 145 cervical cancer patients in order to determine their suitability to predict survival. Progression-free survival (PFS) was the primary endpoint, and overall survival (OS), pelvic PFS (PPFS), and distant metastasis-free survival (DMFS) were the secondary endpoints. Blood samples were collected before and 1 month after radiotherapy to measure serum biomarker levels. ApoC-II was measured using a monoclonal antibody-based enzyme-linked immunosorbent assay, which was developed for this purpose. Kaplan-Meier method, log-rank test, and univariate and multivariate Cox proportional hazards models were used for statistical analyses. In multivariate analysis, larger tumor size was independently associated with shorter PFS, OS, PPFS, and DMFS, while longer overall treatment time was independently associated with shorter PPFS. Higher pretreatment SCC-Ag (*P* < 0.001) was associated with shorter DMFS. Higher posttreatment SCC-Ag (*P* = 0.017) was also associated with shorter DMFS. Pretreatment ApoC-II was associated with PPFS in univariate analysis (*P* = 0.048), but not in multivariate analysis. Patients with pretreatment ApoC-II levels ≤ 25.8 μg/ml had shorter PPFS than those with pretreatment ApoC-II levels > 25.8 μg/ml (*P* = 0.023, log-rank test). Pre- and posttreatment serum SCC-Ag and pretreatment serum ApoC-II levels may be important biomarkers to predict survival outcomes of patients with cervical cancer after radiotherapy. Pre- and posttreatment SCC-Ag and pretreatment ApoC-II might be useful in clinical settings for screening patients to improve treatment strategies in cervical cancer.

## Introduction

Cervical cancer (CC) is the third most common gynecologic malignancy and the fourth leading cause of death in women worldwide, with an estimated global incidence of 604,000 new cases in 2020 [1]. Currently, the standard treatment for locally advanced CC is definitive concurrent chemoradiotherapy (CCRT) [2], which results in a 3-year disease-free survival (DFS) of 65%–75% [2,3]. Unfortunately, 30%–50% patients develop local recurrence or distant metastatic disease after radiotherapy (RT) [3] and approximately 90% of patients with distant recurrence die within 5 years due to the disease [4]. Thus, recurrence of CC remains a major problem in CC patients, and identifying prognostic factors in patients with locally advanced CC treated with RT is very important. However, until now, no definitive tumor marker has been available to predict tumor prognosis in CC after RT.

Serum is a favorable sample type due to its low cost and easy accessibility. Serum biomarkers are important tools in cancer management. Previously, we have used surface-enhanced laser desorption and ionization-time-of-flight mass spectrometry to identify serum protein biomarkers that had different protein expression patterns in healthy women and patients with CC. As a result, we identified apolipoprotein C-II (ApoC-II) as a potential predictor of post-RT outcomes in patients with CC [5]. ApoC-II with a mass of 8.9 kDa, is a small protein that is mainly synthesized in the liver and secreted in the plasma as a surface component of triglyceride-rich lipoproteins (TRLs) [6]. ApoC-II acts as a cofactor of lipoprotein lipase, the main enzyme that hydrolyses plasma triglycerides on TRLs, and thus plays a critical role in TRL metabolism [7]. Low-density lipoproteins shrink after the release of lipids, and ApoC-II either reenters the circulation or is utilized by cancer cells [8]. In this prospective multicenter cohort study, we validated the usefulness of ApoC-II for the prediction of survival outcomes after RT in patients with advanced CC.

Matrix metalloproteinases (MMPs), as members of the zinc-dependent protease family, are involved in proteolysis and degradation of the extracellular matrix [9]. MMPs can be synthesized not only by tumor cells but also by surrounding stromal cells. MMP1 has been shown to play a key role via the epithelial mesenchymal transition in the regulation of cervical tumor growth and lymph node metastasis [10]. Indeed, previous studies have revealed enhanced expression of MMP2 in CC lesions, as well as a strong association of MMP2 with the development of metastases leading to poor clinical outcome in CC patients [11]. In our previous study, we used cDNA microarrays and identified MMP1 as one of the genes associated with progression and metastasis of advanced CC after radiotherapy [12]. Recently, Chen et al. reported that ApoC-II knockdown suppressed MMP2 expression [13]. Interestingly, ApoC-II has been demonstrated to be cleaved by MMPs [14]. Hence, in the current study we evaluated serum levels of MMP1 and MMP2 in relation to serum ApoC-II as potential prognostic biomarkers for CC.

Serum squamous cell carcinoma antigen (SCC-Ag), a widely known biomarker for CC, is created through the formation of epithelium of the uterine cervix squamous cells and presents higher expression along with neoplastic transformation of the cervical squamous epithelium [15]. The 99th percentile of circulating SCC-Ag has been shown to be 1.9 μg/l in healthy women [16], and serum SCC-Ag level is upregulated in 28–88% of patients with squamous cell CC [17]. SCC-Ag shows high sensitivity for initial diagnosis of squamous cell CC compared with tissue polypeptide antigen, carcinoembryonic antigen, and cancer antigen 125 [17]. However, the clinical relevance of SCC-Ag in the management of CC remains controversial. Some previous studies have reported that SCC-Ag evaluation during follow-up did not improve early detection of the recurrence. On the contrary, other authors described that increased SCC-Ag levels were related to the stage of disease, tumor size, depth of the stroma, and lymphadenopathies [18]. Further evaluation is needed to determine the practical usefulness of SCC-Ag in the treatment strategy for CC.

In this prospective multicenter cohort study, we evaluated the usefulness of ApoC-II (a previously reported candidate [5]), MMP1, MMP2, and SCC-Ag, as prognostic serum biomarkers for patients with CC treated with RT.

## Materials and methods

### Patients

This multicenter prospective study was conducted at 13 institutions by the Japanese Radiation Oncology Study Group (JROSG) between March 2012 and July 2016 in accordance with the principles of the Declaration of Helsinki. The study protocol was approved by the Kansai Medical University Review Board. All participants signed an informed consent form that was approved by the Institutional Review Board of each center.

The eligibility criteria included: histologically proven squamous cell carcinoma of the uterine cervix; International Federation of Gynecology and Obstetrics (FIGO, 2009) stage IB–IVA [19]; Eastern Cooperative Oncology Group (ECOG) performance status, 0–2; age 20–85 years; no para-aortic lymph node metastasis; no history of radiotherapy, chemotherapy, or surgery for CC; and written informed consent.

The exclusion criteria included: cervical stump carcinoma; active synchronous or metachronous (< 5 years) double cancers; pregnancy or lactation; trying to conceive; uncontrolled concurrent medical or neurologic conditions; serious complications affecting treatment, including connective tissue disorders; uncontrolled diabetes; serious, chronic heart failure or cerebrovascular disorder in the 3 months prior to study enrollment; active infection; pacemaker; and ineligibility based on the investigator’s assessment.

Tumor staging was performed by radiation oncology and gynecologic oncology staff according to the FIGO classification.

### Treatment

Whole pelvic external beam RT (EBRT) was administered using 6- or 10-MV high-energy linear accelerators. The radiation was delivered to the tumor in fractions of 1.8–2 Gy daily, 5 days per week, with a standard 4-technique. An additional dose was given to the parametria with central shielding, 4 times per week. The patients also received ^192^Ir high-dose-rate intracavitary brachytherapy (HDR-ICBT). The ^192^Ir brachytherapy was given to point A (2 cm lateral to the central canal of the uterus and 2 cm above the mucous membrane of the lateral fornix in the axis of the uterus) [20] at a dose of 5–6 Gy per session, once per week, 3–4 times. Additionally, patients were treated weekly with cisplatin, at a dose of 30–40 mg/m^2^ for 3–5 cycles. Intensity-modulated radiotherapy (IMRT) and 3D image-guided brachytherapy (3D-IGBT) were not permitted in this study.

### Follow-up and endpoints

Follow-up of the patients was done every month for the first year after treatment termination, every 2–3 months for the following 4 years, and every 6 months thereafter. Diagnosis of local failure, lymph node metastasis, and distant metastasis was based on physical examination, blood chemistry profile, chest radiograph, computed tomography (CT) scans, magnetic resonance imaging (MRI), 18F-fluorodeoxyglucose positron emission tomography/CT (PET/CT), and biopsy.

Survival of the patient was determined as the interval from the start of radiotherapy to the occurrence of outcome events or censor. The primary endpoint was progression-free survival (PFS), defined as the interval from the start of radiotherapy to pelvic progression, distant metastasis, or death due to CC. Secondary endpoints included overall survival (OS), pelvic PFS (PPFS), and distant metastasis-free survival (DMFS).

Local failure was defined as tumor persistence or reappearance at the primary site in cervix or in pelvis including lymph nodes in the radiation field. Pelvic lymph node metastases were determined with or without biopsy when both of the following two criteria were met: 1) spherical swollen lymph nodes larger than 10 mm on CT or MRI; 2) abnormal accumulation or hot spot of FDG on PET/CT. Distant metastasis was diagnosed when metastatic tumor lesions outside the pelvis were detected by CT, MRI, or PET/CT, inspection, or palpation with or without biopsy, which included hematogenous metastasis such as lung, liver, or bone metastasis; lymph node metastasis beyond regional lymph nodes such as para-aortic, supraclavicular, or inguinal lymph nodes metastasis; vaginal or vulvar metastasis; and cancerous ascites or pleural effusion.

### Collection of serum samples and measurements of SCC-Ag, MMP1, and MMP2

Fasting serum samples were collected at the initial visit and 1 month after the end of radiotherapy. Approximately 6 ml of blood was drawn by venipuncture and the blood was put in the VENOJECT^®^II Blood Collecting Tubes containing gel for serum separation (Terumo Corporation, Tokyo, Japan) and then placed on ice for 30 min. The blood was then centrifuged at 3,000 rpm for 20 min, and the serum was aliquoted approximately 1 ml in the polyethylene test tube and stored at -80°C until use. Samples were analyzed sequentially. Multiple freeze-thaw cycles were avoided. The serum SCC-Ag level was measured using an IMx SCC microparticle enzyme immunoassay kit (Abbott Laboratories, Abbott Park, IL, USA) according to the manufacturer’s instructions. MMP1 Kit (Z-7P) and MMP2 Kit (Z-8) (KYOWA PHARMA CHEMICAL CO., LTD., Tokyo, Japan) were used to measure serum levels of MMP1 and MMP2, respectively [21,22].

### Production and purification of monoclonal antibodies to human apolipoprotein-CII

We developed a new sensitive ELISA system to measure serum ApoC-II levels (Japanese patent application no. 5366234/2013, METHOD FOR PROGNOSIS AFTER RADIATION THERAPY FOR SQUAMOUS CELL CERVICAL CANCER AND KIT FOR PROGNOSIS). We injected 50 μL of human ApoC-II (hApoC-II) protein from human plasma (Sigma-Aldrich Corp. St. Louis, MO, USA) and complete Freund’s adjuvant at a 1:1 ratio into BALB/c mice at 1, 4, and 7 days. Lymphocytes were isolated from immunized mice and fused with P3U1 myeloma cells at a 5:1 ratio using the polyethylene glycol 4000 method 3 days after the final injection. Hybridoma supernatants were screened for binding ability by an indirect ELISA using immunizing antigen.

Briefly, hApoC-II protein was immobilized on 96-well immunomodule plate (Nalgen Nunc International, Rochester, NY, USA) in 50uL PBS at 4°C overnight. The plates were blocked with 200 μl PBS containing 1.0% BSA and 0.09% NaN3 at 4°C overnight. Hybridoma supernatants was added to each well and incubated for 1 h at room temperature, followed by washing with PBS. The bound antibodies were detected by Anti-IgG (H+L chain) (Mouse) pAb-HRP (Medical and Biological Laboratories Co., Ltd. Nagano, Japan). After 3 washes, the wells were incubated in substrate solution (Medical and Biological Laboratories Co., Ltd. Nagano, Japan) for 15 min. The reaction was stopped by the addition of a 1.5 M phosphoric acid solution. A spectrophotometer at a wavelength of 450 nm with a reference wavelength of 620 nm was used to determine the color intensity. Selected positive hybridoma colonies (ELISA OD value ≥0.2) were expanded and subcloned by limiting dilution. An isostrip kit (Hoffmann-La Roche, Basel, Switzerland) was used for antibody isotype determination according to the manufacturer’s instructions. Antibody purification was carried out with Protein G-Sepharose column chromatography (GE Healthcare, Buckinghamshire, UK).Biotinylation of clone 47–4 was performed by using EZ-Link NHS-LC-Biotin (Pierce, Rockford, IL, USA) according to the manufacturer’s instructions. The mAbs were produced according to the investigator’s instruction by Medical and Biological Laboratories Co., Ltd. (Nagano, Japan).

### Measurement of serum levels of human apolipoprotein-CII

Serum levels of human ApoC-II were measured by a standard sandwich ELISA using two purified hApoCII mAbs, clone 72–1 (IgG3κ) and clone 47–4 (IgG2bκ). Briefly, a 96-well immunomodule microplate (Nalgen Nunc International, Rochester, NY, USA) was precoated with 10μg/mL hApoC-II mAb (clone 72–1) in 100uL PBS by overnight incubation at 4°C, followed by incubation with a blocking buffer (PBS/1% BSA/ 0.09% NaN_3_) for 2 h at room temperature. Following coating, 1,000-fold diluted sera were added to the wells and incubated for 1 h. The plates were washed 5 times with 0.05% Tween-PBS to remove any unbound hApoC-II, and 0.25ug/mL the biotinylated anti-hApoC-II mAb (clone 47–4) was added to the wells. After incubation for 1 h, the wells were washed 5 times with 0.05% Tween-PBS, followed by incubation with a 100,000-fold diluted horseradish peroxidase-labeled streptavidin antibody (BIOSOURCE, Camarino, CA, USA) for 1 h. After 5 washes, the wells were incubated in tetramethylbenzidine substrate solution (Moss Inc. Pasadena, ML, USA) for 30 min. The reaction was stopped by the addition of a 0.18-M sulfuric acid solution. A spectrophotometer at a wavelength of 450 nm with a reference wavelength of 620 nm was used to determine the color intensity. A typical standard curve of the ELISA is presented in S1 Fig. Positive or negative controls could not be set for the assessment with ELISA. Serum ApoC-II were detected and quantified for all serum samples.

### Statistical analysis

Patients who could not complete the definitive radiation therapy and those who were lost to follow-up within 1 year of the observation period was excluded from the statistical analysis. Patient characteristics and clinical variables were summarized as medians (range) for continuous variables, and as numbers (%) for categorical variables. Associations between clinical variables (i.e., pre- or posttreatment serum biomarkers, patient characteristics, and treatment information) and survival outcomes were evaluated using univariate Cox proportional hazards models. The proportional hazards assumption in Cox models was confirmed using the Schoenfeld residuals. Hazard ratios (HRs) with 95% confidence intervals (CIs) and *P*-values were calculated. Time-dependent receiver operating characteristic (ROC) [23] analysis was conducted for serum biomarkers that showed significance in the univariate model, and the area under the ROC curve (AUC) was calculated. The optimal cut-off value was determined based on the Youden index [24], and the sensitivity and specificity were calculated to predict survival outcome. Patients were stratified into two groups according to the cut-off value, and the cumulative survival rate was estimated and compared between groups using the Kaplan-Meier method and log-rank test. Variables that showed *P*-value <0.1 in the univariate analyses were further analyzed in the multivariate Cox proportional hazards models. Because the outcome events were sparse, Firth’s bias correction method [25] was applied to the Cox models to address complete separation. Spearman’s rank correlation coefficient was used to assess multicollinearity among independent variables. Backward stepwise selection method was used, and the best-fit model was determined based on Akaike information criteria (AIC) for Firth’s method [26]. In addition, correlation between serum biomarkers and clinical variables was evaluated using Spearman’s rank correlation coefficient. Statistical analyses were performed using IBM SPSS Statistics 23.0 (IBM Corporation, Armonk, New York, United States) and R version 3.3.2 (http://www.R-project.org; R Foundation for Statistical Computing, Vienna, Austria). A two-tailed *P*-value below 0.05 was considered significant.

## Results

### Patient characteristics

In total, 148 patients with CC were enrolled between March 2012 and September 2014 and followed up until July 2016, with the exception of 3 patients who were excluded before treatment due to ineligibility. Of 145 eligible patients, 3 were excluded from statistical analysis; 1 died of pneumothorax before completing definitive radiotherapy and 2 transferred to another hospital within 1 year of the observation period. The clinical characteristics and serum levels of pre- and posttreatment biomarkers of 142 analyzed patients are shown in Table 1. Most patients (82.4%) were treated weekly with cisplatin at a total dose of 40–350 mg/m^2^ for 1–7 cycles. The cumulative linear quadratic equivalent dose (EQD2) was 64.0 (41.3–90.0) Gy, and was prescribed at point A. The serum ApoC-II level significantly increased after RT (*P* < 0.001).

**Table 1:** Patient characteristics (n = 145) and clinical information.

Parameters		Median (range), n (%)
Age (years)		59 (23–85)
Tumor size (cm)		5.0 (1.4–13.0)
PS	0	113 (79.6)
	1	27 (19.0)
	2	2 (1.4)
BMI		21.8 (15.1–35.4)
Hb		12.0 (6.0–17.2)
FIGO stage	Ib1	17 (12.0)
	Ib2	18 (12.7)
	IIa	6 (4.2)
	IIb	48 (33.8)
	IIIa	6 (4.2)
	IIIb	41 (28.9)
	IVa	6 (4.2)
Pelvic lymph node adenopathy on image examination	Negative	88 (62.0)
	Positive	54 (38.0)
External radiotherapy (Gy)	Whole-pelvis	36.0 (20.0–50.0)
	Central shielding	16.0 (9.0–45.0)
High-dose rate to point A (Gy)		21.4 (6.0–30.0)
EQD2		64.0 (41.3–90.0)
Overall treatment time (days)		47 (35–91)
Chemotherapy	No	25 (17.6)
	Yes	117 (82.4)
Serum biomarkers: Pretreatment		
SCC-Ag (ng/mL)		6.6 (0.5–144.0)
MMP1 (ng/mL)		13.4 (1.8–105.0)
MMP2 (ng/mL)		747 (453–1430)
ApoC-II (μg/mL)		23.0 (6.7–62.8)
Serum biomarkers: Posttreatment		
SCC-Ag (ng/mL)		1.0 (0.5–5.3)
MMP1 (ng/mL)		10.3 (2.2–88.0)
MMP2 (ng/mL)		983.5 (542–1730)
ApoC-II (μg/mL)		29.4 (9.0–90.7)

Abbreviations: PS, performance status; BMI, body mass index; FIGO, International Federation of Gynecology and Obstetrics; EQD2, equivalent dose in 2Gy fractions; SCC-Ag, squamous cell carcinoma antigen; MMP1, matrix metalloproteinase-1; MMP2, matrix metalloproteinase-2; ApoC-II, apolipoprotein C-II.

**Fig 1:**
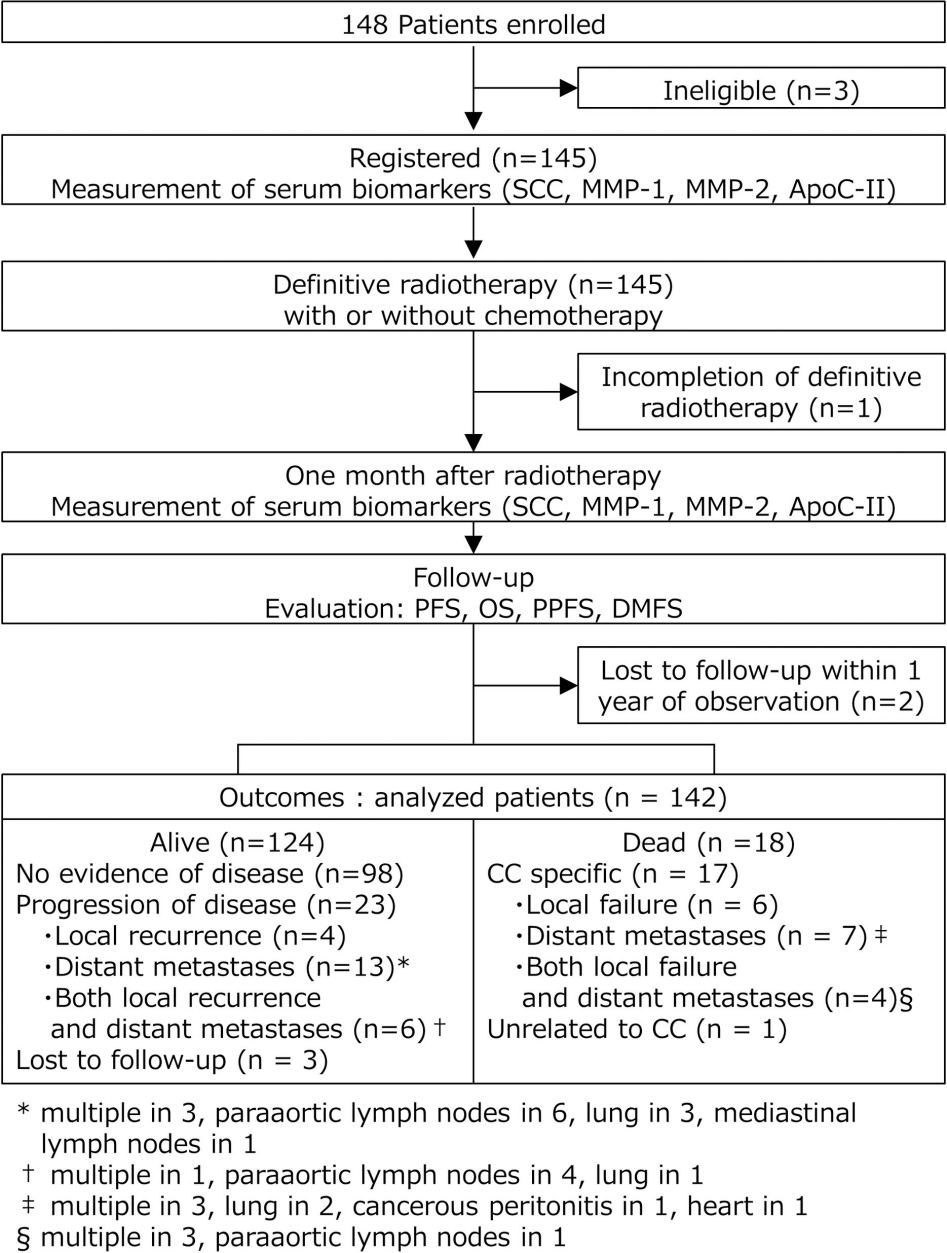
Patient disposition and clinical outcome. Of 148 enrolled, 3 were excluded before starting the treatment, 1 could not complete definitive radiotherapy, and two transferred to another hospital with less than 1 year of observation. Of 142 analyzed, 98 were alive with no evidence of disease, 23 were alive with progression of disease, 18 were dead, and 3 were lost to follow-up during the observation period.

Fig 1 depicts disposition outcome of patients. The median follow-up period of the 142 analyzed patients was 28.4 (range, 2.8–52.7) months. Patients who developed recurrence were further treated with RT and/or CCRT after completion of the initial treatment.

### Primary endpoint: Progression-free survival

In the univariate Cox models, performance status (PS; *P* = 0.034), tumor size (*P* < 0.001), FIGO stage (*P* = 0.004), pelvic lymph node adenopathy (*P* = 0.048), overall treatment time of radiation therapy (*P* = 0.019), and pretreatment SCC-Ag (*P* < 0.001) were significantly associated with PFS (Table 2).

**Table 2:** Pre-and posttreatment biomarkers and clinical parameters Univariate analysis for PFS, OS, PPFS, and DMFS.

	PFS			OS			PPFS			DMFS		
	HR	95% CI	*P*	HR	95% CI	*P*	HR	95% CI	*P*	HR	95% CI	*P*
Age _per10	1.068	.862–1.329	.548	1.146	.832–1.602	.408	1.003	.744–1.358	.984	1.073	.835–1.386	.582
BMI	.921	.839–1.001	.054	.959	.841–1.072	.488	.968	.857–1.075	.569	.914	.821–1.006	.066
Hb	.901	.767–1.067	.222	.806	.645–1.029	.082	.887	.712–1.123	.310	.928	.767–1.134	.458
PS≥1	2.094	1.062–3.929	.034*	1.781	.602–4.575	.275	1.386	.478–3.430	.519	2.218	1.013–4.576	.047*
Tumor size	1.630	1.347–1.963	< .001*	2.172	1.645–2.959	< .001*	1.634	1.266–2.076	< .001*	1.498	1.189–1.876	.001*
LN_positive	1.848	1.005–3.407	.048*	2.580	1.042–6.785	.040*	1.529	.651–3.554	.323	2.334	1.151–4.839	.019*
FIGO ≥III	2.469	1.345–4.603	.004*	3.094	1.246–8.160	.015*	1.594	.678–3.707	.278	2.636	1.297–5.476	.008*
Chemo	.835	.412–1.895	.644	.574	.223–1.699	.294	.625	.252–1.811	.359	.682	.314–1.668	.379
OTT _per10	1.612	1.088–2.251	.019*	1.505	.942–2.109	.081	2.010	1.261–2.958	.005*	1.515	.924–2.277	.095
EQD2	.990	.940–1.043	.708	1.003	.925–1.087	.947	.978	.909–1.054	.566	.989	.931–1.053	.740
HDR point A	.981	.905–1.065	.639	1.074	.951–1.216	.253	.966	.865–1.085	.563	1.005	.916–1.106	.914
Serum biomarkers, pretreatment												
SCC _per10	1.186	1.089–1.275	< .001*	1.185	1.045–1.312	.011*	1.016	.831–1.166	.851	1.260	1.154–1.363	< .001*
MMP1 _per10	1.104	.944–1.246	.194	1.152	.905–1.364	.214	1.186	.988–1.362	.065	1.000	.779–1.189	.997
MMP2 _per100	.944	.785–1.121	.520	.968	.730–1.249	.813	.827	.621–1.071	.155	.894	.715–1.098	.294
ApoC2 _per10	1.026	.744–1.368	.870	.725	.402–1.199	.226	.606	.346-.995	.048*	1.126	.780–1.553	.509
Serum biomarkers, posttreatment												
SCC	1.441	.952–1.988	.080	1.157	.512–1.900	.668	.645	.219–1.397	.325	1.689	1.113–2.333	.017*
MMP1 _per10	1.189	.960–1.393	.103	1.038	.598–1.384	.854	1.198	.854–1.487	.245	1.170	.910–1.399	.191
MMP2 _per100	1.074	.918–1.238	.360	1.116	.881–1.374	.347	1.032	.818–1.268	.783	1.075	.896–1.265	.424
ApoC2 _per10	.910	.714–1.128	.407	.701	.435–1.030	.073	.714	.462–1.015	.062	.977	.747–1.236	.858

*P < .05.

Abbreviations: PFS, progression-free survival; OS, overall survival; PPFS, pelvic progression-free survival; DMFS, distant metastasis-free survival; HDR, high-dose rate; HR, hazard ration; CI, confidence interval; BMI, body mass index; Hb, hemoglobin; PS, performance status; LN, pelvic lymph node adenopathy; FIGO, International Federation of Gynecology and Obstetrics; Chemo, chemotherapy; EQD2, equivalent dose in 2Gy fractions; SCC, squamous cell carcinoma antigen; MMP1, matrix metalloproteinase-1; MMP2, matrix metalloproteinase-2; ApoC-II, apolipoprotein C-II.

**Fig 2:**
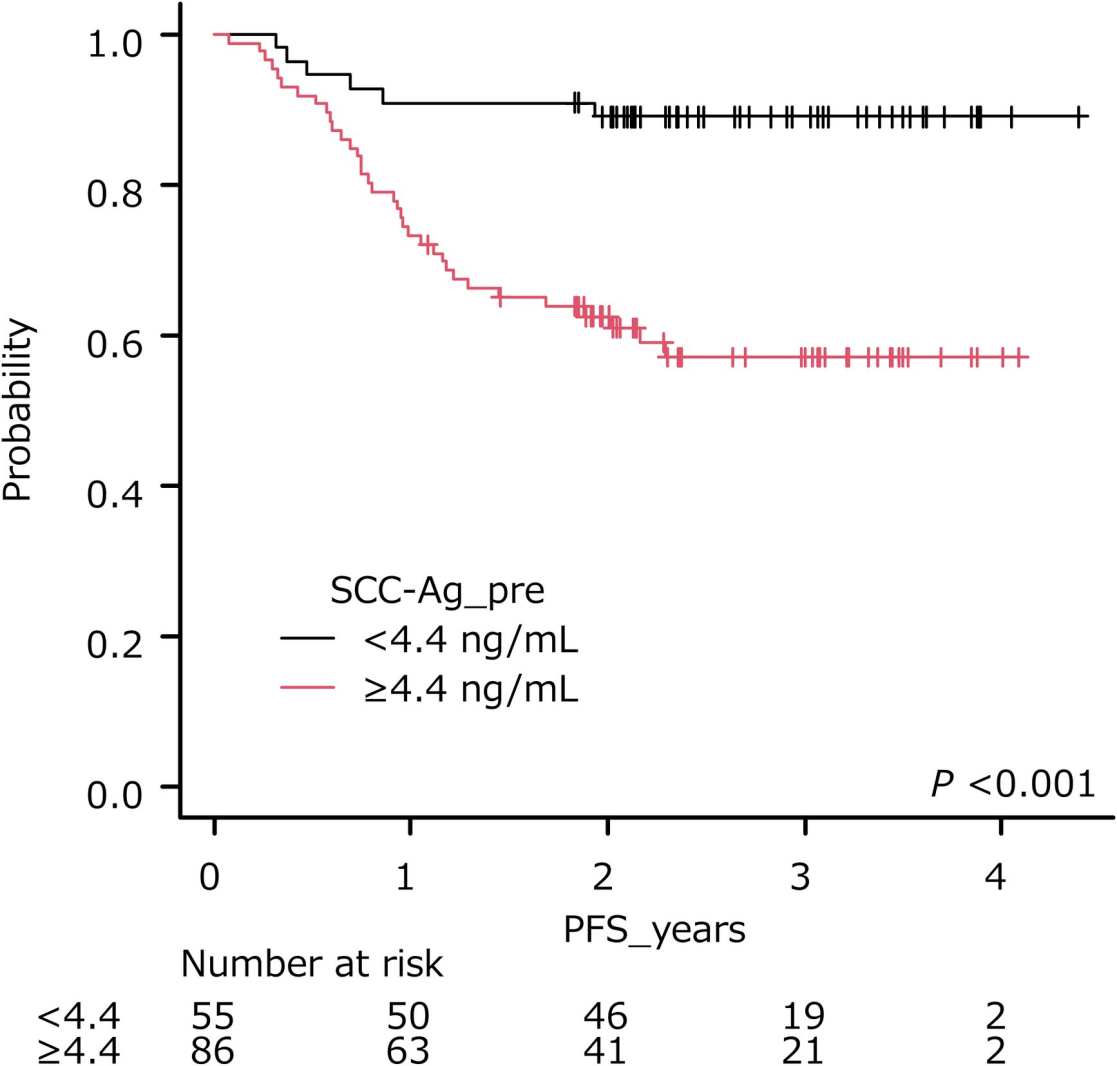
Progression-free survival of patients stratified by pretreatment SCC levels. Higher levels of pretreatment SCC-Ag (≥4.4 ng/ml) were significantly associated with poorer outcome (*P* < 0.001).

The optimal cut-off of SCC-Ag was 4.4 ng/ml (AUC, 0.705; sensitivity, 0.869; specificity, 0.502: Table 3), and the cumulative survival rate of patients stratified by the cut-off is shown in Fig 2. Patients with pretreatment SCC-Ag levels ≥ 4.4 ng/ml had a shorter PFS than those with pretreatment SCC-Ag levels < 4.4 ng/ml (*P* < 0.001, log-rank test).

**Table 3:** Results of time-dependent ROC analysis for significant variables in univariate Cox proportional hazard models.

	PFS			OS			PPFS			DMFS		
	cut-off	AUC	Se, Sp	cut-off	AUC	Se, Sp	cut-off	AUC	Se, Sp	cut-off	AUC	Se, Sp
PS≥1		.580	.316, .844								.579	.325, .833
Tumor size	≥4.2	.710	.946, .389	≥4.7	.774	.917, .509	≥4.2	.685	.955, .332	≥4.2	.681	.975, .370
LN(+)		.583	.496, .669		.635	.609, .661					.609	.547, .671
FIGO≥III		.637	.565, .709		.588	.522, .654					.623	.561, .685
OTT	≥49	.604	.539, .688				≥49	.672	.670, .671			
Serum biomarkers, pretreatment												
SCC-Ag	≥4.4	.705	.869, .502	≥4.4	.725	.967, .455				≥13.2	.739	.632, .778
ApoC2							<25.8	.631	.856, .400			
Serum biomarkers, posttreatment												
SCC-Ag										≥2.0	.593	.249, .928

Abbreviations: ROC, receiver operating characteristic; PFS, progression-free survival; OS, overall survival; PPFS, pelvic progression-free survival; DMFS, distant metastasis-free survival; AUC, area under the ROC curve; Se, sensitivity; Sp, specificity; PS, performance status; LN, pelvic lymph node adenopathy; FIGO, International Federation of Gynecology and Obstetrics; OTT, overall treatment time; SCC-Ag, squamous cell carcinoma antigen; MMP1, matrix metalloproteinase-1; MMP2, matrix metalloproteinase-2; ApoC-II, apolipoprotein C-II.

Multivariate analysis suggested that tumor size (*P* < 0.001) and pretreatment SCC-Ag levels (*P* = 0.033) were independent predictors for PFS. Regarding serum biomarkers, higher SCC-Ag levels were associated with shorter PFS (Table 4).

**Table 4:** Pre-and posttreatment biomarkers and clinical parameters best-fit model in Multivariate analysis for PFS, OS, PPFS, and DMFS.

	PFS			OS			PPFS			DMFS		
	HR	95% CI	*P*	HR	95% CI	*P*	HR	95% CI	*P*	HR	95% CI	*P*
Clinical parameters and pretreatment serum biomarkers												
Tumor size	1.527	1.240–1.863	< .001*	2.176	1.649–2.959	< .001*	1.697	1.309–2.174	< .001*	1.328	1.030–1.696	.029*
OTT_per10	1.446	.983–2.020	.060				1.837	1.144–2.738	.014*			
SCC-Ag_per10	1.120	1.010–1.223	.033*							1.219	1.104–1.328	< .001*
Clinical parameters and posttreatment serum biomarkers												
BMI	.929	.841–1.016	.110							.917	.821–1.011	.083
Tumor size	1.533	1.247–1.885	< .001*	2.184	1.561–3.130	< .001*	1.527	1.122–2.091	.007*	1.461	1.157–1.846	.002*
OTT_per10	1.399	.936–1.971	.097				1.712	1.052–2.571	.032*			
SCC-Ag										1.596	1.035–2.235	.036*
ApoC2_per10				.730	.489-.983	.037*	.783	.522–1.056	.119			

*P < .05.

Abbreviations: PFS, progression-free survival; OS, overall survival; PPFS, pelvic progression-free survival; DMFS, distant metastasis-free survival; HR, hazard ration; CI, confidence interval; OTT, overall treatment time; BMI, body mass index; SCC-Ag, squamous cell carcinoma antigen; BMI, body mass index; ApoC-II, apolipoprotein C-II.

### Secondary endpoint: Overall survival

In the univariate Cox models, tumor size (*P* < 0.001), pelvic lymph node adenopathy (*P* = 0.040), FIGO stage (*P* = 0.015), and pretreatment SCC-Ag (*P* = 0.011) were significantly associated with OS (Table 2).

**Fig 3:**
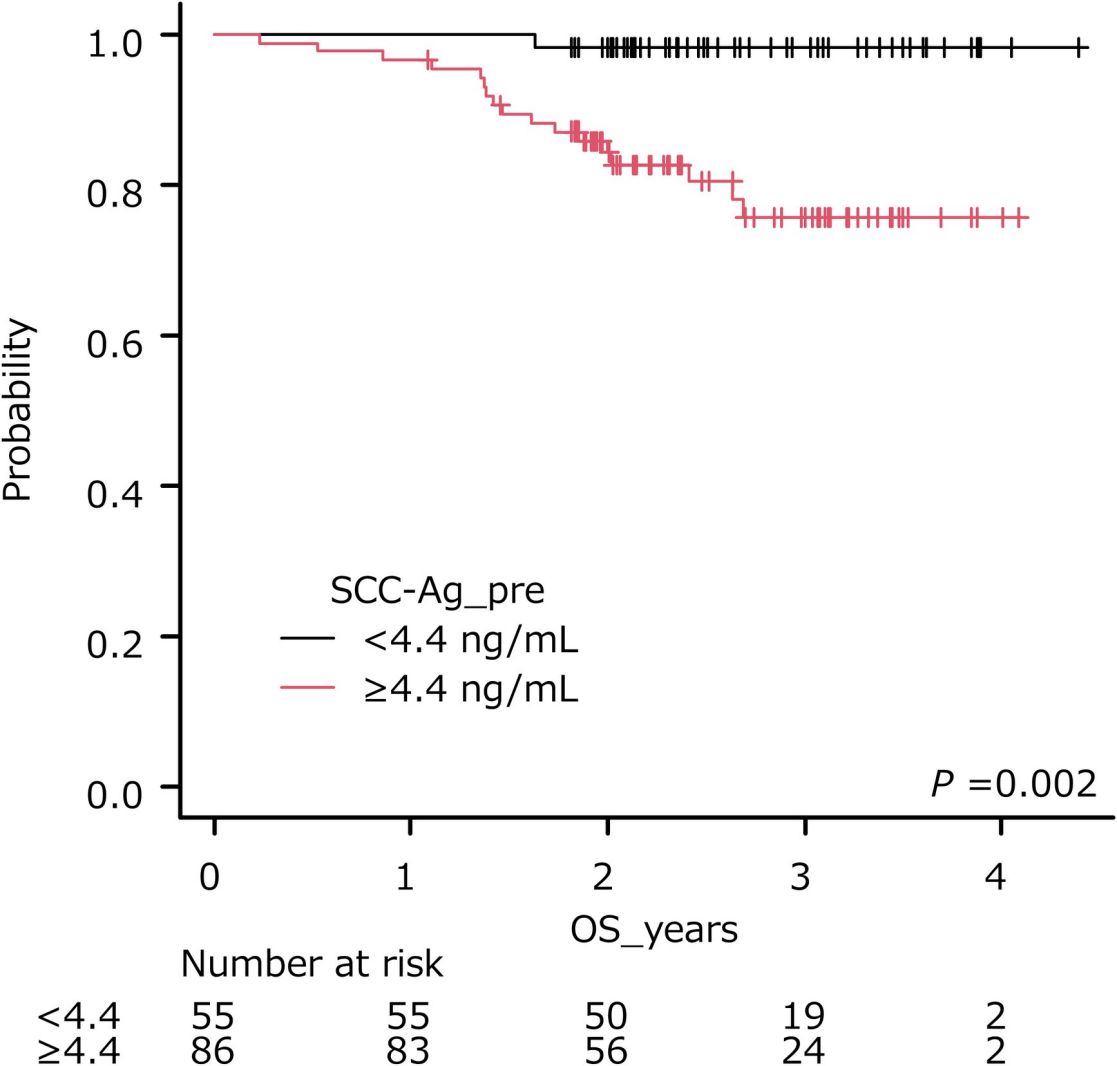
Overall survival of patients stratified by pretreatment SCC levels. Higher levels of pretreatment SCC-Ag (>4.4 ng/ml) were significantly associated with poorer outcome (*P* = 0.002).

The optimal cut-off of SCC-Ag was 4.4 ng/ml (AUC, 0.725; sensitivity, 0.967; specificity, 0.455: Table 3). The cumulative survival rate of patients stratified by the cut-off is shown in Fig 3. Patients with pretreatment SCC-Ag levels ≥ 4.4 ng/ml had a shorter OS than those with pretreatment SCC-Ag levels < 4.4 ng/ml (*P* = 0.002, log-rank test).

Multivariate analysis suggested that tumor size was an independent predictor for OS in that larger tumor size was associated with a shorter OS (HR, 2.176; 95% CI, 1.649–2.959; *P* < 0.001).

Multivariate analysis for posttreatment biomarkers suggested that tumor size and posttreatment ApoC-II were independent predictors for OS. Regarding serum biomarkers, lower ApoC-II (HR, 0.730; 95% CI, 0.489–0.983; *P* = 0.037) were associated with shorter OS (Table 4).

### Secondary endpoint: Pelvic progression-free survival

In the univariate Cox models, tumor size (*P* < 0.001), overall treatment time (*P* = 0.005), and pretreatment ApoC-II (*P* = 0.048) were significantly associated with PPFS (Table 2).

**Fig 4:**
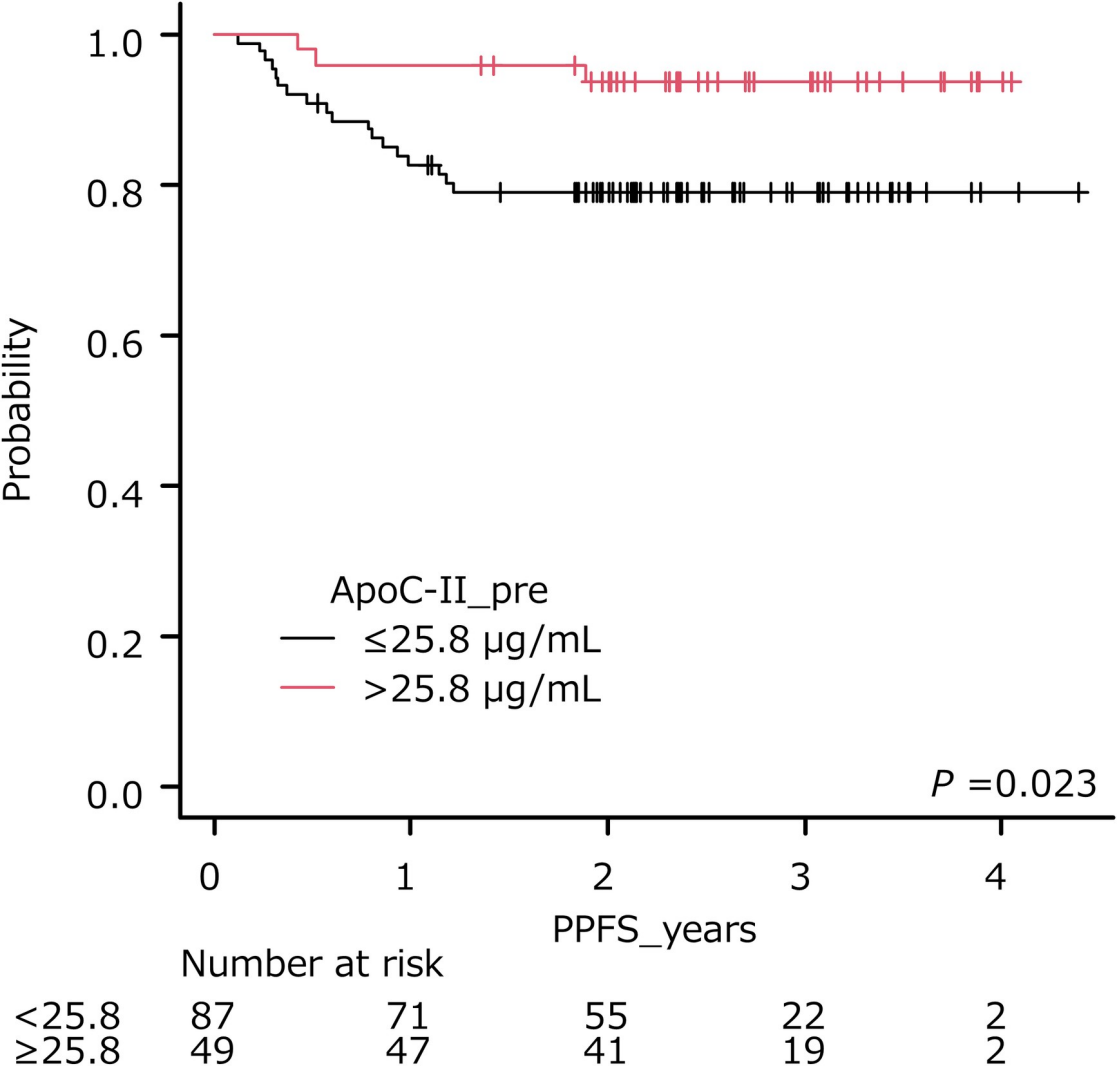
Pelvic progression-free survival of patients stratified by pretreatment ApoC-II levels. Lower levels of pretreatment ApoC-II (≤25.8 μg/ml) were significantly associated with poorer outcome (*P* = 0.023).

The optimal cut-off of ApoC-II was 25.8 μg/ml (AUC, 0.631; sensitivity, 0.856; specificity, 0.400) (Table 3). The cumulative survival rate of patients stratified by the cut-off is shown in Fig 4. Patients with pretreatment ApoC-II levels ≤ 25.8 μg/ml had a shorter PPFS than those with pretreatment ApoC-II levels > 25.8 μg/ml (*P* = 0.023, log-rank test).

Multivariate analysis suggested that tumor size and overall treatment time were independent predictors for PPFS. Larger tumor size (HR, 1.697; 95% CI, 1.309–2.174; *P* < 0.001) and longer overall treatment time (HR, 1.837; 95% CI, 1.144–2.738; *P* = 0.014) were associated with shorter PPFS.

### Secondary endpoint: Distant metastasis-free survival

In the univariate Cox models, PS, tumor size, pelvic lymph node adenopathy, FIGO stage, and pre- and posttreatment SCC-Ag were significantly associated with DMFS (Table 2).

**Fig 5:**
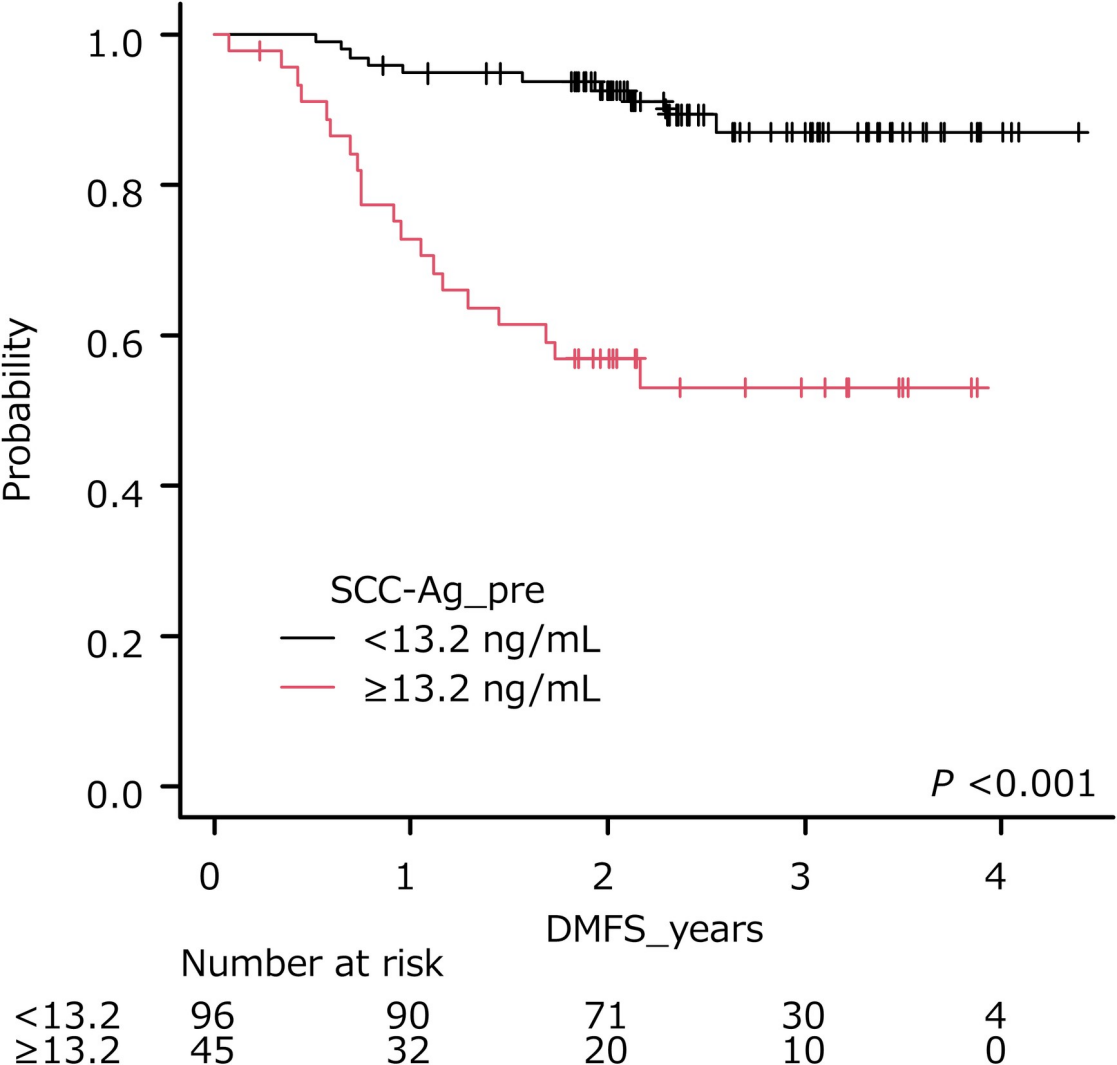
Distant metastasis-free survival of patients stratified by pretreatment SCC-Ag levels. Higher levels of pretreatment SCC-Ag (≥13.2 ng/ml) were significantly associated with poorer outcome (*P* < 0.001).

**Fig 6:**
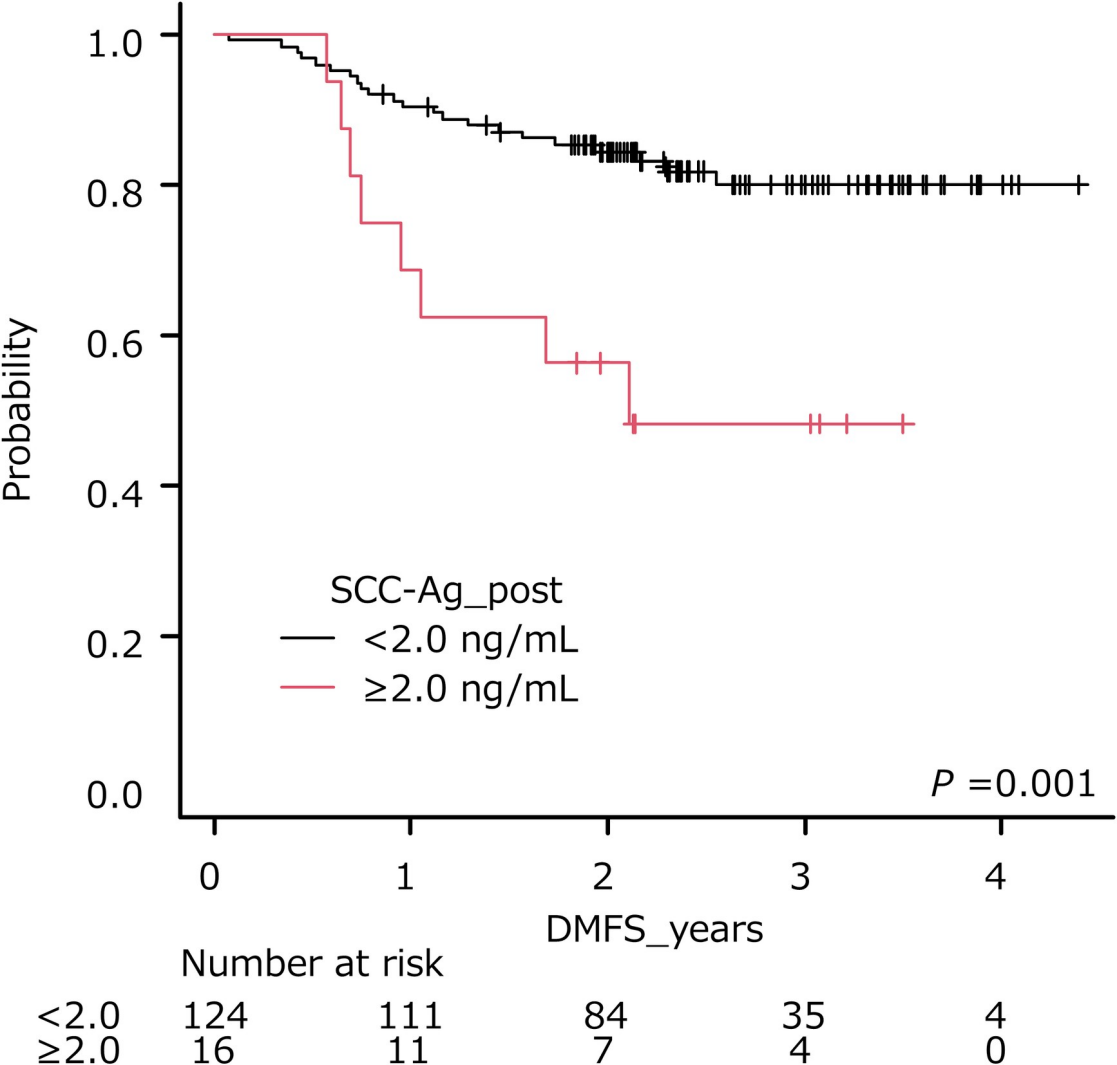
Distant metastasis-free survival of patients stratified by posttreatment SCC-Ag levels. Higher levels of pretreatment SCC-Ag (≥2.0 ng/ml) were significantly associated with poorer outcome (*P* = 0.001).

The optimal cut-off of pre- and posttreatment SCC-Ag was 13.2 ng/ml (AUC, 0.739; sensitivity, 0.632; specificity, 0.778) (Table 3) and 2.0 ng/ml (AUC, 0.593; sensitivity, 0.249; specificity, 0.928), respectively. The cumulative survival rate of patients stratified by each cut-off is shown in Figs 5 and 6. Patients with pretreatment SCC-Ag levels ≥ 13.2 ng/ml had a shorter DMFS than those with pretreatment SCC-Ag levels < 13.2 ng/ml (*P* < 0.001, log-rank test), while patients with posttreatment SCC-Ag levels ≥ 2.0 ng/ml had a shorter DMFS than those with posttreatment SCC-Ag levels < 2.0 ng/ml (*P* = 0.001, log-rank test).

Multivariate analysis suggested that tumor size and pretreatment SCC-Ag were independent predictors for DMFS. Larger tumor size (HR, 1.328; 95% CI, 1.030–1.696; *P* = 0.029) and higher pretreatment SCC-Ag (HR, 1.219; 95% CI, 1.104–1.328; *P* < 0.001) were associated with shorter DMFS (Table 4).

Multivariate analysis for posttreatment biomarkers suggested that tumor size and posttreatment SCC-Ag were independent predictors for DMFS. Regarding serum biomarkers, higher posttreatment SCC-Ag (HR, 1.596; 95% CI, 1.035–2.235; *P* = 0.036) were associated with shorter DMFS (Table 4).

### Correlation analysis

The correlation between pretreatment serum biomarkers and clinical variables is shown in Table 5. Pretreatment SCC-Ag, but not ApoC-II, was positively correlated with tumor size (*P* < 0.001).

**Table 5:** The correlation between pretreatment serum biomarkers and clinical variables.

		SCC-Ag	MMP1	MMP2	ApoC-II
age	Coef	.065	-.152	.280	.302
	*P*	.442	.071	.001*	< .001*
PS	Coef	.074	.197	.112	.003
	*P*	.382	.019*	.186	.976
BMI	Coef	-.201	-.226	.036	.295
	*P*	.017*	.007*	.674	< .001*
FIGO	Coef	.322	.114	-.201	.088
	*P*	< .001*	.176	.017*	.309
tumor size	Coef	.515	.206	-.226	-.115
	*P*	< .001*	.014*	.007*	.182
LN	Coef	.288	.132	.020	-.077
	*P*	.001*	.120	.814	.372
Hb	Coef	-.396	-.261	.079	.353
	*P*	< .001*	.002*	.352	< .001*

*P < .05.

Abbreviations: SCC-Ag, squamous cell carcinoma antigen; MMP1, matrix metalloproteinase-1; MMP2, matrix metalloproteinase-2; ApoC-II, apolipoprotein C-II; PS, performance status; BMI, body mass index; FIGO, International Federation of Gynecology and Obstetrics; LN, pelvic lymph node adenopathy; Hb, hemoglobin.

## Discussion

Beyond stratification factors, such as FIGO stage and regional lymph node involvement [27], the outcomes after RT are heterogeneous; thus, additional reliable biomarkers need to be validated and incorporated into clinical practice. Although several studies have investigated the usefulness of serum biomarkers, including carcinoembryonic antigen [28], CYFRA 21–1 [29], microRNAs [30], and circulating tumor cells [31], for the prognosis of CC patients after RT, as yet there are no established biomarkers. Therefore, in order to identify serum biomarkers, including the previously reported ApoC-II, for survival after RT for CC, we conducted this multicenter prospective study at 13 institutions in Japan.

In this study, pretreatment ApoC-II was associated with PPFS in Cox univariate analysis, which verified our previous findings [5]. Patients with pretreatment ApoC-II levels ≤ 25.8 μg/ml had a shorter PPFS than those with pretreatment ApoC-II levels > 25.8 μg/ml. Although previous studies described correlations between serum ApoC-II expression and the risk of occurrence or progression in cancer, including pancreatic cancer [8], colorectal cancer [32], and breast cancer [33], to the best of our knowledge, this is the first report to provide the prognostic value of the serum ApoC-II level in CC treated with RT.

The major apolipoproteins include ApoAs (ApoA-I, ApoA-II, ApoA-IV), ApoB, ApoCs (ApoC-I, ApoC-II, ApoC-III), and ApoE. The regulation of lipoprotein metabolism requires specific apolipoproteins as cofactors for enzymes of lipid metabolism, concerning their involvement in the transport and redistribution of lipids among various cells and tissues or their maintenance of lipoprotein particles structure [34]. Some studies have investigated the correlations between serum lipid levels and human cancers [35,36]. Significant upregulation of lipid metabolism is seen during carcinogenesis, due to mostly upregulated or activated lipogenic enzymes in cancer cells [37]. Lipids also facilitate cancer development, invasion, and metastasis, because highly proliferative malignant cells usually show strong lipid avidity, increasing either the endogenous synthesis or the exogenous uptake of lipids [38].

The clinical importance of serum ApoC-II as a useful predictor for cancer survival is currently unknown. Xue et al. showed that an increased level of pretreatment serum ApoC-II was significantly associated with poorer survival in patients with pancreatic cancer after surgery [8]. However, our findings indicate that a decreased level of pretreatment serum ApoC-II was significantly associated with poorer PPFS in patients with CC treated with RT, regardless of the tumor size. Long-term prospective follow-up evaluation will be warranted to determine the clinical value of serum ApoC-II in CC treated with RT.

In the present study, the optimal cut-off of pretreatment SCC-Ag level for PFS or OS was at 4.4 ng/ml, and the difference in PFS and OS was statistically significant between the patient groups stratified by cut-off. Choi et al. reviewed patients with cervical squamous cell carcinoma treated with CCRT and found that the recurrence-free survival (RFS) rates of those with pretreatment SCC-Ag levels < 4 ng/ml and ≥ 4 ng/ml were 80.2% and 56.6% (*P* < 0.001), respectively [39]. In the current study, patients with pretreatment SCC-Ag levels ≥ 13.2 ng/ml had a shorter DMFS compared to those with pretreatment SCC-Ag levels < 13.2 ng/ml. In previous reports, patients with elevated levels of SCC-Ag were associated with high rates of distant metastases [28,39]. Indeed, Huang et al. reported that pretreatment SCC-Ag levels ≥ 10 ng/ml were associated with para-aortic lymph node relapse after CCRT Revised FIGO staging for carcinoma of the cervix. [28], while Choi et al. reported that pretreatment SCC-Ag levels ≥ 4 ng/ml was related to 3-year distant metastasis [39]. In a study by Kang et al., the pretreatment SCC-Ag level was an independent prognostic factor of distant recurrence, and was utilized to predict distant recurrence within 5 years [40].

In the current study, the optimal cut-off of posttreatment SCC-Ag (1 month after treatment completion) for DMFS was 2.0 ng/ml, and higher posttreatment SCC-Ag was associated with shorter DMFS. In a study of 783 patients with CC, Ryu et al. reported that the optimal cut-off point for the posttreatment SCC-Ag level to predict recurrence was 0.9 ng/ml (sensitivity 44.2%, specificity 72.0%), while the posttreatment SCC-Ag level was also independently associated with DFS (*P* = 0.003) [41]. Kawaguchi et al. reviewed patients with CC treated with definitive RT or CCRT, and demonstrated that in patients with posttreatment SCC-Ag levels (1 month after the completion of treatment) < 1.15 ng/ml and ≥ 1.15 ng/ml, the 3-year OS rates were 90.7% and 36.6% (*P* < 0.001), and the 3-year PFS were 74.7% and 19.5% (*P* < 0.001), respectively [42]. Olsen et al. reported that CC patients who could not achieve normalized posttreatment SCC-Ag levels (< 2.2 ng/ml, at treatment completion) after CCRT was accompanied by an incomplete metabolic response on PET/CT imaging after 3-month of treatment and showed a decreased PFS [43]. Although the cut-off points varied, that is, 0.9 ng/ml, 1.15 ng/ml, and 2.2 ng/ml (2.0 ng/ml in our study), the survival rates of patients with elevated posttreatment SCC-Ag levels were poor [40–42]. Furthermore, according to a meta-analysis, posttreatment SCC-Ag level is a predictor of recurrence and survival for CC patients treated with RT, CCRT, or surgery [44]. These results indicate that adjuvant chemotherapy [45], additional brachytherapy, salvage hysterectomy, or other currently available adjuvant therapies are relevant in these cases. Even though no definitive evidence has been established that these adjuvant therapies are effective in improving outcomes for CC patients, they are needed for patients with a high risk of tumor recurrence.

In this study, there was no association between serum levels of MMP1, MMP2, and the prognosis of CC patients with RT. Although there have been several reports on serum MMP1 in the context of exploring biomarkers for human cancers [46–48], according to our knowledge, no previous study has evaluated serum MMP1 as a prognostic marker in CC patients after RT. Braicu et al. [49] analyzed the role of biomarkers, including serum MMP2, in monitoring the response to adjuvant CCRT using serum from 72 patients with CC, and concluded that serum MMP2 had no predictive value, in agreement with our results.

Tumor size is known as a strong prognostic clinical factor. Our study showed that large tumor size was a significant factor of poor PFS (≥ 4.2 cm), OS (≥ 4.7 cm), PPFS (≥ 4.2 cm), and DFS (≥ 4.2 cm) in Cox univariate and multivariate analysis; these results were in accordance with previous reports that cervical tumor size was an independent prognostic factor for both recurrence and survival [39,50]. In a study by Perez et al. [50], larger tumor size (< 3 cm vs. 3–5 cm vs. > 5 cm) was significantly associated with poorer RFS in FIGO Ib to III CC. Choi et al. also demonstrated that a tumor size larger than 4.5 cm was associated with shorter RFS in FIGO Ib to IVa CC [39].

In our study, pretreatment SCC-Ag levels were associated with tumor size. While the pathological stages of CC progresses, the infiltration depth becomes deeper, the tumor size becomes larger, and SCC-Ag comes to enter the tumor lymphatics, likely resulting in intrusion of the tumor cells into the bloodstream. As more cancer antigens are produced and released into the serum, more elevated levels of SCC-Ag are found in the venous blood of CC patients [51]. However, there was no correlation between pretreatment ApoC-II levels and tumor size in our study. Further prospective and large-sample studies are needed to clarify the prognostic value and the action mechanisms of serum ApoC-II in CC.

There are some limitations in our study. First, the median follow-up duration was relatively short at 28.4 months, leading to a lower incidence of outcome events than expected, which might have caused some overlooking of potentially significant factors in statistical analysis due to a low statistical power. Second, IMRT and 3D image-guided brachytherapy (3D-IGBT) were not performed in this study because the treatment schedule for EBRT and HDR-ICBT was primarily used in Japan during this study period. Third, negative or positive controls could not be set for ApoC-II ELISA, although serum ApoC-II levels were successfully quantified for all serum samples of the patients.

## Conclusions

Pre- and posttreatment SCC-Ag and pretreatment serum ApoC-II levels are potentially useful biomarkers for prediction of prognosis in locally advanced CC patients after RT. However, these findings might be valid only on a Japanese population. Studies involving larger CC patient cohorts combining other populations with long-term evaluation of survival outcomes may be required for further verification of these findings. Pretreatment serum biomarker approaches provide benefit to both clinicians and patients with accessible, non-invasive sample collection procedures, and facilitate personalized therapy to identify optimal treatment options and clinical strategy improvements for the treatment of locally advanced CC.

## Supporting information

S1 Fig Typical standard curve for the apolipoprotein C-II ELISA. (TIF)

S1 File TREND checklist. (DOC)

S2 File The clinical trial protocol_Japanese. (DOCX)

S3 File The main points of clinical study protocol_English. (DOCX)

S4 File ApoC_trial_minimal dataset. (XLSX)
