# Supplementary figures and images for: Clinical value of serum biomarkers, squamous cell carcinoma antigen and apolipoprotein C-II in follow-up of patients with locally advanced cervical squamous cell carcinoma treated with radiation: A multicenter prospective cohort study
Source: PLoS One. 2021 Nov 2;16(11):e0259235. doi: 10.1371/journal.pone.0259235 (PMC8562853; doi:10.1371/journal.pone.0259235)

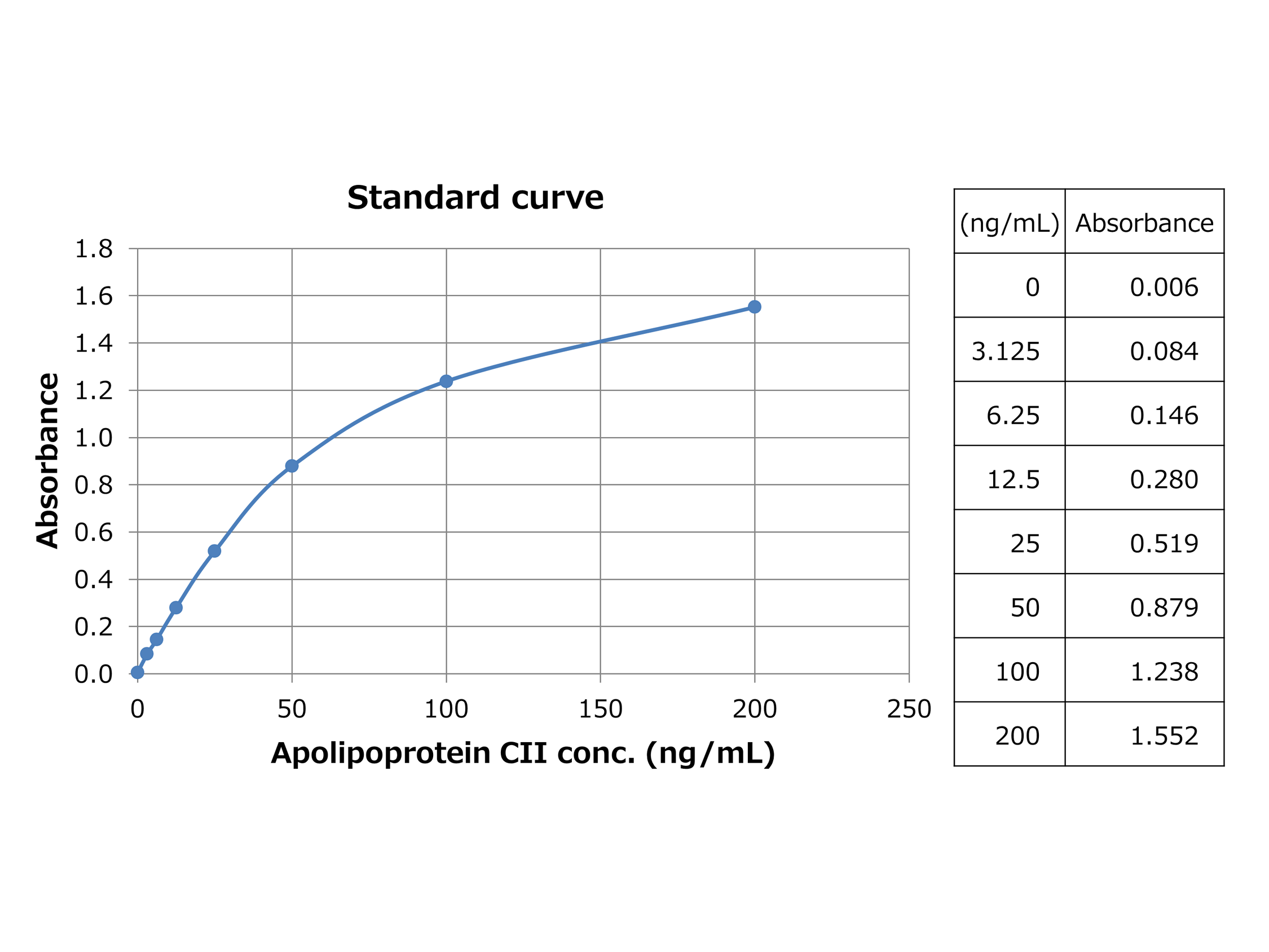

Supplement: S1 Fig — (TIF) [file pone.0259235.s001.tif]
