# Supplementary material for: Clinical value of serum biomarkers, squamous cell carcinoma antigen and apolipoprotein C-II in follow-up of patients with locally advanced cervical squamous cell carcinoma treated with radiation: A multicenter prospective cohort study
Source: PLoS One. 2021 Nov 2;16(11):e0259235. doi: 10.1371/journal.pone.0259235 (PMC8562853; doi:10.1371/journal.pone.0259235)
Supplement: S2 File — (DOCX) [file pone.0259235.s003.docx]

***JROSG***

**子宮頸癌の放射線治療予後予測因子としての**

**バイオマーカー（ApoC-II）の再現性評価に関する**

**多施設共同前向き試験**

**実施計画書**

***ＪＲＯＳＧ 10-4***

**試験タイプ：**前向き試験

**研究代表者**

関西医科大学附属滝井病院放射線科　播磨洋子

〒570-8507　大阪府守口市文園町10-15

TEL: 06-6992-1001

FAX: 06-6993-3865

E-mail: harima@takii.kmu.ac.jp

**研究事務局**

関西医科大学附属滝井病院放射線科　播磨洋子

〒570-8507　大阪府守口市文園町10-15

TEL: 06-6992-1001

FAX: 06-6993-3865

E-mail: harima@takii.kmu.ac.jp

**データセンター**

近土写真製版株式会社メディカル事業部　伊東俊幸

住所：〒543-0011大阪市天王寺区清水谷町11-15

TEL: 0120-619-198

FAX: 0120-219-199

E-mail: ito@mydo-kond.co.jp

平成23年7月30日 提出版

平成23年8月19日 第二版

平成23年１2月2１日 第三版

**（試験タイトル）　子宮頸癌の放射線治療予後予測因子としてのバイオマーカー（ApoC-II）の再現性評価**

**（研究の分類）　前向きコホート研究およびネステッド・ケース・コントロール研究**^1), 2)^

**0. 概要**

**0.1. シェーマ**

血液採取

2年観察

放射線治療（標準治療）

登録

子宮頸部扁平上皮癌患者

FIGO Stage Iｂ～IVa期

ECOG PS 0～2

20歳以上85歳以下

登録期間に参加施設を訪れた子宮頸癌患者のうち，選択基準を満たす症例を前向きコホート研究に登録し，標準治療を施した後，背景を治療者にマスクして経過観察を実施する．

また，イベント発生例（増悪・全死など）をケース，それに年齢（±5歳）と病期でマッチング（ケース ： コントロール ＝ 1 : 1）させたイベント非発生例をコントロールとしてネステッド・ケース・コントロール研究を行い，アポリポ蛋白C-II（ApoC-II）等のバイオマーカー値を比較する．

**0.2. 目的**

関西医科大学放射線科学講座において子宮頸癌患者血液を用いて放射線治療予後予測因子とし

てアポリポ蛋白C-II（ApoC-II）を同定し，モノクロナール抗体Elisa-Kitを作成した．本研究の目的

は，子宮頸癌の放射線治療後の予後が初診時の血清ApoC-IIで予測できるか否かを検証するため

に，多施設共同前向き研究を行うことである．ApoC-IIを測定するために前向き研究であることが必

要となる．

1）プライマリーエンドポイント：無増悪生存期間

2）セカンダリーエンドポイント：全生存期間，骨盤内無増悪生存期間，遠隔転移無発生生存期間

**0.3. 対象**

以下の選択基準を満たす子宮頸癌患者を対象とする．

＜適格基準＞

1. 原発巣が子宮頸部扁平上皮癌であることが組織学的に確認されている患者
2. FIGO臨床病期(1994年) がIb～Ⅳa期の患者
3. 一般状態(ECOG Performance Status, PS)が0～2の患者
4. 登録時の年齢が20歳以上85歳以下の患者
5. 傍大動脈リンパ節転移陰性の患者
6. 子宮頸癌に対して初回治療の患者
7. 本試験の参加について十分な説明を受けて趣旨を理解した上で文書に同意した患者

＜除外基準＞

1. 断端癌患者
2. 活動性の重複癌を有する患者．ここでは，同時性重複癌及び無病期間が5年以内の異時性重複癌をいう．ただし，局所療法により治癒と判断されるCarcinoma in Situは活動性の重複癌には含めない．
3. 妊娠中，妊娠の可能性がある患者，もしくは授乳中である患者．
4. 挙児を希望している患者
5. 精神病または精神症状を合併しており試験への参加が困難な患者
6. 治療の実施に支障を来すと判断される重篤な合併症（膠原病・制御困難の糖尿病）を有する患者
7. 3ヶ月以内の心疾患の既往がある患者，もしくは慢性心不全の患者
8. 3ヶ月以内の重篤な脳血管障害の既往がある患者
9. 活動性の感染症を有する患者
10. 心臓ペースメーカーをしている患者
11. HBs抗原が陽性の患者
12. その他，臨床試験担当医師が本試験の対象として不適当と判断した患者

**0.4. 治療内容**

放射線治療は原発巣に対して強い腫瘍制御能力が見込まれる．よって，可能な限り放射線治療が

スケジュール通りに完遂されることを優先する．また，本研究の目的は，子宮頸癌を対象に放射線治

療予後因子を同定するために，患者血液を用いて抽出したApoC-IIを検証するための多施設共同

前向き試験を行うことであるので，化学療法は必ずしも施行しなくても許容される．

外部照射と高線量率腔内照射(HDR-ICBT)を組み合わせた放射線治療を実施する．

外部照射は全骨盤照射50-50.4Gy/25-28回（中央遮蔽30-41.4Gyより），HDR-ICBTはA点線量，

週1回で6Gy，計3回から4回で18Gy-24Gyを基本とする．

同時化学放射線療法を施行する場合はCDDP 30～40 mg/m^2^（最大量70mg/body）を週１回静脈内

投与，1回投与を1コースとし，合計3～5コース投与．**0.5. 予定登録者数と研究期間**

予定登録数は150例とする．

登録期間は12ヶ月（1年），追跡期間は登録後24ヶ月（2年間）で総研究期間は36ヶ月（3年間）と

する．

**0.6. 問い合わせ先**

適格基準など：研究事務局

関西医科大学　滝井病院　放射線科　准教授　播磨洋子

住所：大阪府守口市文園町10-15

Tel: 06-6992-1001

Fax: 06-6993-3865

E-mail: harima@takii.kmu.ac.jp

登録手続き：データセンター

近土写真製版株式会社メディカル事業部　伊東俊幸

住所：〒543-0011大阪市天王寺区清水谷町11-15

Tel: 0120-619-198

Fax: 0120-219-199

E-mail: ito@mydo-kond.co.jp

目次

[**0. 概要** 2](#_Toc266691473)

[**0.1. シェーマ** 2](#_Toc266691474)

[**0.2. 目的** 2](#_Toc266691475)

[**0.3. 対象** 3](#_Toc266691476)

[**0.4. 治療内容** 3](#_Toc266691477)

[**0.5. 予定登録者数と研究期間** 4](#_Toc266691479)

[**0.6. 問い合わせ先** 4](#_Toc266691480)

[**1. 目的とエンドポイント** 7](#_Toc266691481)

[**1.1. 目的** 7](#_Toc266691482)

[**1.2. エンドポイント** 7](#_Toc266691483)

[**2. 背景と試験計画の根拠** 7](#_Toc266691484)

[**2.1. 背景** 7](#_Toc266691485)

[**2.2. 対象**](#_Toc266691486) 8

[**2.3. 対象疾患に対する標準的治療**](#_Toc266691487) 9

[**2.4. 試験デザインと根拠** 1](#_Toc266691488)2

[**2.5. 試験参加に伴って予測される利益と危険（不利益）** 12](#_Toc266691489)

[**2.6. 本試験の意義** 12](#_Toc266691490)

[**3. 本試験で用いる基準・定義** 13](#_Toc266691491)

[**3.1. 病期診断基準** 13](#_Toc266691492)

[**3.2. 予後判定基準** 14](#_Toc266691493)

[**4. 症例基準** 15](#_Toc266691494)

[**4.1. 適格基準** 15](#_Toc266691495)

[**4.2. 除外基準** 15](#_Toc266691495)

[**4.3. 休止・再開基準** 15](#_Toc266691495)

[**4.4. 中止基準** 15](#_Toc266691495)6

[**4.5. 脱落基準** 15](#_Toc266691496)6

[**5. 登録** 16](#_Toc266691497)

[**6. 実施方法** 17](#_Toc266691500)

[**7. スタディカレンダー（検査日程）** 17](#_Toc266691501)

[**8. データ収集** 20](#_Toc266691502)

[**8.1. 記録用紙の種類と提出期限** 20](#_Toc266691503)

[**8.2. 調査用紙の送付方法** 20](#_Toc266691504)

[**9. 有害事象** 20](#_Toc266691505)

[**9.1. 有害事象の評価** 20](#_Toc266691506)

[**9.2. 予想される有害事象** 20](#_Toc266691507)

[**9.3. 有害事象発生時の対応** 20](#_Toc266691507)

[**10. 効果判定とエンドポイントの定義**](#_Toc266691508) 20

[**10.1.　効果判定**](#_Toc266691509) 20

[**10.2. エンドポイント** 21](#_Toc266691510)

[**11. 統計的事項** 21](#_Toc266691512)

[**11.1. 中間解析と試験の早期中止** 21](#_Toc266691515)

[**11.2. 最終解析** 21](#_Toc266691516)

[**12.　倫理的事項** 22](#_Toc266691517)

[**12.1.　患者の保護** 22](#_Toc266691518)

[**12.2.　インフォームド・コンセント** 22](#_Toc266691519)

[**12.3.　プライバシーの保護と患者識別** 24](#_Toc266691520)

[**12.4.　プロトコールの遵守** 24](#_Toc266691521)

[**13. 研究費用および利益相反** 24](#_Toc266691522)

[**13.1. 研究費用** 24](#_Toc266691523)

[**13.2. 利益相反** 25](#_Toc266691524)

[**14. モニタリングと監査** 24](#_Toc266691522)

[**14.1. 定期モニタリング** 24](#_Toc266691523)

[**14.2. 施設訪問監査** 25](#_Toc266691524)

[**15.　研究組織** 25](#_Toc266691525)

[**15.1.　研究代表者** 25](#_Toc266691526)

[**15.2.　参加施設および研究連携者** 25](#_Toc266691527)

[**15.3.　データセンター** 25](#_Toc266691528)

[**15.4.　プロトコール作成責任者** 25](#_Toc266691528)

[**15.5.　質的管理委員会** 26](#_Toc266691529)

[**16.　研究成果の発表** 26](#_Toc266691530)

[**17.　参考文献** 27](#_Toc266691531)

[**18.　付表Appendix** 27](file:///C:\Users\Harima\Desktop\submt%2020201012\AppData\Local\Microsoft\Windows\Temporary%20Internet%20Files\Content.Outlook\AppData\Local\Microsoft\Windows\Temporary%20Internet%20Files\多田一人\デスクトップ\播磨先生\播磨先生より2010年7月15日受領_現在作業中\修正済みプロトコル.doc#_Toc267035710)

**1）説明文書・同意書**

**2）ヘルシンキ宣言（日本医師会和訳）**

**3）ECOG のPerformance Status（PS）の日本語訳**

**4）記録用紙（CRF）**

**・登録票**

**・登録確認票**

**・調査票**

**5）有害事象共通用語基準 v4.0日本語訳JCOG版**

**1. 目的とエンドポイント**

**1.1. 目的**

放射線治療に対する応答に個体差が存在し，個々の症例で治療効果は明らかに異なる．そこで進行期子宮頸癌の治療成績を向上させるためには，放射線抵抗性に関与する遺伝子や分子を追求し，治療戦略を確立する以外に方法はない．

本研究の目的は進行期子宮頸癌を対象に放射線治療予後予測因子を同定するために、患者血清

を用いて抽出したApoC-IIを検証するための多施設共同前向き研究を行うことである．

matrix Metalloproteinase（MMP）は子宮頸癌の進行・浸潤に関与したと報告され^5)^，予後不良群

の治療前の血清でMMPが高発現するとApoC-IIを切断し， ApoC-IIの低発現を認めるとの仮説を

立て，ApoC-IIとMMPに注目して証明する．また，子宮頸部扁平上皮癌患者におけるSCC値は予

後予測因子として一定していないので，本研究で検討する．治療前と治療終了後1ヶ月に各々6ml

の採血により，ApoC-II，matrix metalloproteinase-1（MMP1），matrix metalloproteinase-2

（MMP2），SCCを測定する．

**1.2. エンドポイント**

1）プライマリーエンドポイント：無増悪生存期間

2）セカンダリーエンドポイント：全生存期間，骨盤内無増悪生存期間，遠隔転移無発生生存期間

**2. 背景と試験計画の根拠**

**2.1. 背景**

バイオマーカーとは，アメリカ食品医薬品局（FDA）により「正常な生物学的過程，発病の過程，もしく

は治療介入による薬理学的反応を反映する測定，および評価可能な特性」と定義されている．癌バ

イオマーカーは癌細胞自身，もしくは発癌に応じて変化する細胞，組織に由来する分子であり，癌

の発見，分類，診断，予後予測，治療効果の確認等のさまざまな目的で利用されている．癌の組織

型の違いにより扁平上皮癌のSCC抗原，腺癌のCEA抗原，発症臓器の違いにより前立腺癌のPSA

抗原がある．しかし，子宮頸癌の臓器特異的な指標はなく，また放射線治療効果に関与するバイオ

マーカーは未だ判明していない．播磨らは平成19 年～21 年度科学研究費補助金基盤研究(B)

「分子生物学的手法を用いた子宮頸癌の放射線治療効果予測因子の探索と検証」により，放射線

治療前の患者血清を用いてプロテインチップでバイオマーカー侯補蛋白としてアポリポ蛋白C-II

（ApoC-II）を同定し，モノクロナール抗体によるApoC-II Elisaキットを作成した（特許申請中：特願

2008-214992）．Elisa (Enzyme-linked immunosorbent assay) は，特異性の高い抗原抗体反応を利

用し，微量タンパク質の検出・定量に広く用いられている．播磨らは作成したApoC-II Elisaキットが

子宮頸癌の放射線治療予後を予測できるか否かを検討するために，2003年1月から2009年10月

までに関西医科大学で放射線治療を施行した65例の子宮頸部扁平上皮癌を対象として，ApoC-II

の原病生存率への関与を観察した．予後良好群は治療後無再発生存を確認した35例（平均生

存期間27.8ヶ月）で，不良群は原病死を確認した30例（平均生存期間16.6ヶ月）であった．年齢の

平均61歳，腫瘍径の平均5.9cm，予後良好群の病期はI期2例，II期9例，III期22例，IV期2

例であった．また，不良群の病期はI期1例，II期2例，III期21例，IV期6例であった．初診時の

血清を用いてSCC，ApoC-II，MMP1，MMP2を測定し，初診時癌組織からHPV感染を検索した．

原病生存率をエンドポイントとして，年齢，腫瘍径，病期，HPV，SCC，ApoC-II，MMP1，MMP2と

の相関をCox比例ハザード単変量，多変量で解析した．蛋白発現量はStudent-t testを，特異度，

感度はROC解析を用いた．その結果，予後不良群のApoC-II 発現量は良好群に比べて有意に減

少した(p=0.03）．原病生存率に関与したのは単変量解析ではApoC-II (p=0.01) ，MMP1

(p=0.02) ，病期(p=0.004) ，腫瘍径 (p<0.001)で，多変量解析ではApoC-II (p=0.02) ，病期

(p=0.04) であった．ApoC-IIの特異度は97.9%，感度44.4%であった．この検討結果から，ApoC-II

は予後不良症例に低発現し，ApoC-IIは子宮頸癌の放射線治療予後予測因子として有用なバイオ

マーカーの可能性があると考えられた^3)^．

ApoC-IIは脂肪代謝においてトリグリセリドをグリセロールと遊離脂肪酸に分解する酵素リポプロテインリパーゼを活性化する蛋白である．substrateは触媒である酵素の反応の間，消費される[反応体](http://honyaku.yahoofs.jp/url_result?ctw_=sS,eCR-EJ,bT,uaHR0cDovL2VuLndpa2lwZWRpYS5vcmcvd2lraS9SZWFjdGFudA==,qlang=ja%7Cfor=0%7Csp=-5%7Cfs=100%25%7Cfb=0%7Cfi=0%7Cfc=FF0000%7Cdb=T%7Ceid=CR-EJ,k,t)，または酵素によって作用される分子を示すが，ApoC-IIは酵素MMPのsubstrateで， MMPによって切断されることが分かっている^4)^．

一方， MMPは子宮頸癌の進行・浸潤に関与し^5)^，我々のゲノム解析結果でもMMPが進行・転移に

関与した^6)^．MMPが高発現するとApoC-IIを切断し， ApoC-IIの蛋白量が少なくなるので，予後不

良群の治療前の血清でApoC-IIの低発現を認めたとの仮説を立て， ApoC-IIとMMPに注目して

証明する．また， ApoC-IIが連携研究者の施設においても検証されるか否かについて前向き研究

を行う．

**2.2. 対象**

以下の選択基準を満たす子宮頸癌患者

＜適格基準＞

1. 原発巣が子宮頸部扁平上皮癌であることが組織学的に確認されている患者
2. FIGO臨床病期(1994年) がⅠb～Ⅳa期の患者
3. 一般状態(ECOG Performance Status, PS)が0～2の患者
4. 登録時の年齢が20歳以上85歳以下の患者
5. 傍大動脈リンパ節転移陰性の患者
6. 子宮頸癌に対して初回治療の患者
7. 本試験の参加について十分な説明を受けて趣旨を理解した上で文書に同意した患者

＜除外基準＞

1. 断端癌患者
2. 活動性の重複癌を有する患者．ここでは，同時性重複癌及び無病期間が5年以内の異時性重複癌をいう．ただし，局所療法により治癒と判断されるCarcinoma in Situは活動性の重複癌には含めない．
3. 妊娠中，妊娠の可能性がある患者，もしくは授乳中である患者．
4. 挙児を希望している患者
5. 精神病または精神症状を合併しており試験への参加が困難な患者
6. 治療の実施に支障を来すと判断される重篤な合併症（膠原病・制御困難の糖尿病）を有する患者
7. 3ヶ月以内の心疾患の既往がある患者，もしくは慢性心不全の患者
8. 3ヶ月以内の重篤な脳血管障害の既往がある患者
9. 活動性の感染症を有する患者
10. 心臓ペースメーカーをしている患者
11. HBs抗原が陽性の患者
12. その他，臨床試験担当医師が本試験の対象として不適当と判断した患者

**2.3. 対象疾患に対する標準的治療**

放射線治療は原発巣に対して強い腫瘍制御能力が見込まれる．よって，可能な限り放射線治療が

スケジュール通りに完遂されることを優先する．また，本研究の目的は，子宮頸癌を対象に放射線治

療予後因子を同定するために，患者血液を用いて抽出したApoC-IIを検証するために多施設共同

前向き試験を行うことであるので，化学療法は必ずしも施行しなくても許容される．

**2.3.1. 放射線治療**

以下に示す外部照射と高線量率腔内照射(HDR-ICBT)を組み合わせて治療を行うこととする．もし，

HDR-ICBTが行われなかった場合には理由説明を必要とする．組織内照射は許容しない．放射線

治療の許容総治療期間は8週（56日間）とする．

**2.3.1.1. 外部照射**

放射線治療装置は，6MV以上のX線発生装置で，かつSSD(Source Surface Distance)または

SAD(Source Axis Distance)が100cm以上の装置を用いる．

1）線量と分割法

1日1回1.8または2Gy，週5回法で行う．週4回法は許容されない．表1にわが国における標準放射線治療スケジュール^7)^ を示し，原則としてこのスケジュールにそって放射線治療を行う．

表1 標準放射線治療スケジュール

| 進行期 | 外部照射^*^ | | 腔内照射^#^  HDR (A点線量) |
| --- | --- | --- | --- |
|  | 全骨盤 | 中央遮蔽 |  |
| Ib1, II（小） | 20Gy | 30Gy | 24Gy/4回 |
| Ib2, II（大）, III | 30Gy | 20Gy | 24Gy/4回 |
|  | 40Gy | 10Gy | 18Gy/3回 |
| ＩＶＡ | 40Gy | 10Gy | 18Gy/3回 |
|  | 50Gy | 0Gy | 12Gy/2回 |

HDR: high-dose-rate

*：1回1.8～2Gy，週5回法で行う．画像にて転移が疑われるリンパ節，治療前に結節状に骨盤壁に達する子宮傍組織に対しては，外部照射による追加(boost) 6～10Gyを検討する．

#：1回5～6Gy，週1～2回法で行う．

腔内照射開始後は，外部照射は腔内照射施行日には行わないこととし，その他の日（週4日）に

行う．

2）計画標的体積（PTV）

全骨盤領域をPTVとする．傍大動脈リンパ節領域を含めた，いわゆるextended fieldは許容しな

い．

照射野の形成にはカスタムブロックまたはmultileaf collimatorを用いる．治療は前後対向2門照

射，あるいは直交4門照射にて行う．本研究ではIMRTを許容しない．１回の治療において各門全

ての照射を行う．

①全骨盤照射

－前後照射野

上縁：第5腰椎上縁、または腹部大動脈分岐部

下縁：閉鎖孔下縁，あるいは膣病変の最遠位部位から3cm下方縁

側方縁：小骨盤腔から1.5-2cm外側縁

－側方照射野

上縁/下縁：前後照射野と同一

前縁：恥骨結合前縁から0.5cm前方

後縁：仙骨後縁全体まで含める

②中央遮蔽

原則として全骨盤照射終了以降HDR-ICBT導入時点で，3または4cm幅の中央遮蔽をおく．子宮ゾンデ診が不可能であるなどの技術的理由でHDR-ICBTが開始できない場合，または，技術的にはHDR-ICBT開始可能だが，腫瘍縮小が不十分でさらに外部照射の追加が必要と担当医が判断した場合に限って，中央遮蔽を設置せずにそのまま外部照射を続行することを許可する．

41.4Gy以降も中央遮蔽を挿入しない場合には理由を調査票に記載する．

**2.3.1.2. 高線量率腔内照射(HDR-ICBT)**

放射線治療装置は，リモートアフローダを使用する．タンデムとオボイドを使用し，その種類は問

わない．

1) 開始時期

全骨盤外部照射終了後，1週間以内に開始する．

子宮ゾンデ診が不可能であるなどの技術的理由でHDR-ICBTが開始できない場合，または，技術的にはHDR-ICBT開始可能だが，腫瘍縮小が不十分でさらに外部照射の追加が必要と担当医が判断した場合に限って，50-50.4Gyまで中央遮蔽なしで外部照射を継続した後に再度HDR-ICBTの可能性を判定してもよい．外部照射50-50.4Gy後もHDR-ICBTが技術的に不可能，あるいは不適切と担当医により判断された場合には外部照射によるBoostに切り替える．

2) 線量と分割法

A点線量で規定する．A点の設定は原則として外子宮口を基準とする．

表1のスケジュールにそった線量で1日1回，週1回法，合計3回から5回を行う．表2に外部照射とHDR-ICBTの線量配分例を示す． HDR-ICBT施行日には外部照射を行わない．

なお，50-50.4Gyまで中央遮蔽なしで外部照射を継続した後にHDR-ICBTを行う場合にはHDR-ICBTは11Gy/2回(6Gy+5Gy，5.5Gyx2回)とする(BED 76.5-77.1Gy_10_)．

表2 外部照射とHDR-ICBTの線量配分とBED

| 外部照射  （中央遮蔽なしの線量） | HDR-ICBT  （A点） | 合計　BED*  (Gy_10_) |
| --- | --- | --- |
| 30Gy/15回 or 30.6Gy/17回 | 24Gy/4回 | 74.4 or 74.5 |
| 40Gy/20回 or 41.4Gy/23回 | 18Gy/3回 | 76.8 or 77.8 |

BED=nd[1+d/(α+β)], n=照射回数，

d=1回線量，α/β=10, *外部照射とHDR-ICBTの合計

3) HDR-ICBT施行が不可能と判断された場合

外部照射50-50.4Gy後もHDR-ICBTが技術的に不可能，あるいは不適切と担当医により判断された場合には外部照射によるBoostに切り替える．全骨盤照射のGTVから2-3cmのマージンをとったVolumeに対して，最低64.8Gy(BED76Gy10)，最大72Gy(BED86Gy10)まで投与することとする．その際には直交4門照射などを用い，可及的に小腸を照射野から除外する．

**2.3.2. 化学療法**

**2.3.2.1. 対象**

1)Ib2, IIa2～IVa期を対象とする．

2)腎機能障害がないこと

3)肝機能障害がないこと

4) CDDP投与可能症例

5)次の基準を満たす十分な臓器機能を有するもの

- WBC>3000/m^3^; HGB>10g/dl; Plat>75000/m^3^
- BUN<14mg/dl; CRTN<0.8mg/dl
- GOT<35U/l; GPT<35U/l

**2.3.2.2. 投与スケジュール**

1) CDDPは1週1回投与とし，1回につき30-40mg/m^2^(上限70mg/body)を点滴静注する．放射線

の照射中に同時併用して，計5～6回を施行する．

2) CDDP投与開始は放射線治療開始後出来るだけ速やかに（第1週または第2週）に開始する.

3) 延期は1週間以内とする．

4) 8週間以内に5～6回施行する．

5) CDDPは放射線治療終了後１時間以内に開始し，週１回，計5～6回施行する．

**2.3.2.3. 投与方法**

1) 体表面積から求めたCDDPを生理食塩水（生食）350-500mLに混じ，原則として2時間かけて点滴静注する．

2) CDDP投与前および投与完了後に，1000mL以上の生食などで輸液を行い，十分な時間尿量が得られていることを確認する．

**2.3.3.　後治療**

　　　adjuvant治療を含む後治療に関しては制限を設けない．

**2.4. 試験デザインと根拠**

**2.4.1. 試験デザイン**

　　　前向きコホート研究およびネステッド・ケース・コントロール研究．

登録期間に参加施設を訪れた子宮頸癌患者のうち，選択基準を満たす症例を前向きコホート研究に登録し，血液を採取してApoC-II等のバイオマーカー値を測定する．標準治療を施した後，バイオマーカー値を治療者にマスクした状態で経過観察を実施し，生存時間分析を行う．

また，副次的解析として，イベント発生例（増悪・全死など）をケース，それに年齢（±5歳）と病期でマッチング（ケース ： コントロール ＝ 1 : 1）させたイベント非発生例をコントロールとしてネステッド・ケース・コントロール研究を行い，ApoC-II等のバイオマーカー値を比較する．

予定登録数は150例とし，登録期間は12ヶ月（1年），追跡期間は登録後24ヶ月（2年間）で総研究

期間は36ヶ月（3年間）とする．

**2.4.2. 観察期間の設定根拠**

パイロット研究（子宮頸癌の放射線治療予後予測因子としてのアポリポ蛋白C-II，第48回日本癌治

療学会2010，播磨ら）における再発イベント発生時期が2.3～34.1ヶ月であり，第3四分位が12.2ヶ

月であるところから，第3四分位の2倍である24ヶ月（2年）を観察期間と設定した．

**2.4.3. 登録数設定根拠**

両側有意水準0.05，統計学的パワー80％として，連続変数である曝露要因に中程度の効果量（0.5）^8)^を検出するためには片群64例が必要になる．パイロット研究では65例中30例に再発が見られたことから，この再発率を適用すると，ケース（＝再発例）64例を確保するためには全体で139例のコホートが必要になる．脱落を考慮に入れ，予定登録数を150例とする．

**2.5. 試験参加に伴って予測される利益と危険（不利益）**

本研究においては，資料採取のための治療前後2回の採血以外は標準的な治療を受ける．したが

って本研究に参加する事によって特段の利益または不利益は発生しない．

**2.6. 本試験の意義**

Noordhuisら^9)^ は放射線治療を施行された子宮頸癌患者で，生物学的予後予測因子の有意性をレビューし，COX-2 (cyclooxygenase-2) ^10)^，EGFR (epidermal growth factor receptor) シグナリング（EGFRとC-erbB-2 ^11）^），低酸素（CA9(carbonic anhydrase 9) ^12）^とHIF-1α(hypoxia-inducible factor-1a) ^13)^），血清SCC ^14)^ が治療後予後不良と関係していると報告している．しかし，血清SCCを除いて，局所進行子宮頸癌腫瘍から採取された検体を用いている．血清サンプルの利点は腫瘍検体に比べてより少ない侵襲性で採取可能で，簡便に測定できる点である．

一方，子宮頸部扁平上皮癌患者における血清バイオマーカーについては，SCC，CA 125，VEGF (Vascular endothelial growth factor) が挙げられる^15)^．治療前のSCC値は，子宮頸部扁平上皮癌患者の28-88%で上昇して，腫瘍の病期，腫瘍サイズ，腫瘍の悪性度に相関がある．しかし，SCC値について，予後予測因子として有用ではないとの報告や，生存に関連すると報告もあり一定していない．CA 125は子宮頚部腺癌患者の20-75%で上昇して，病期，腫瘍サイズ，腫瘍の悪性度に関連するが予後との相関は明らかではない．高い血清CA 125値は，子宮頸部扁平上皮癌にも認められたが，低い陽性率である．血清VEGFのレベルは，しばしば子宮頸癌患者で上昇して，成功した治療の後有意に減少するが，血清VEGFと臨床との関連は，まだ検討中である．このように，子宮頸癌患者における有用な予後予測因子としての血清バイオマーカーはいまだ判明していない．

そこで，我々は本研究において局所進行子宮頸癌患者を対象に放射線治療予後因子として血清バイオマーカーApoC-IIを評価することを考えた．

ApoC-IIと癌との関連については膵臓癌^16)^や悪性黒色腫^17)^，白血病^18)^の報告がある．ApoC-IIと関連するMMPは大腸癌^19)^や胃癌^20)^など多くの癌で発現が報告され，とくにHPV感染率が80%以上と高いことが知られている子宮頸癌では，MMP発現率が高い^21)^．このような報告をもとにApoC-IIとMMPの関連に注目して検討する計画をした．

我国において癌死が国民の死因のトップであり，放射線治療機器の進歩とともにQOLを保てる放射線治療の適応となる疾患は増加すると考えられるので，治療効果予測因子システムは有用である．本研究により個々の癌細胞の性質の違い・治療に対する応答性を判別するマーカーの性能を検討し，それらの臨床応用を目指す．

ApoC-IIが高値であれば，治療後の予後が良い可能性があり，将来の研究により放射線線量の軽減や抗癌剤併用投与などの過剰な治療も省くことができるかもしれないので，医療経済の面からも有用であると考えられる．

したがって，本研究は独創的な考えに基づいた研究であり，類似研究はないものと考えられる．

**3. 本試験で用いる基準・定義**

**3.1. 病期診断基準**

病期診断基準にはFIGO子宮頸癌臨床進行期分類 (1994)を用いる．

本試験ではIb～IVa期の患者を対象とする．

0期：浸潤が認められない上皮内癌 (Carcinoma in situ)．

I期：癌が子宮頸部に限局．

Ia期：組織学的に微小浸潤癌が確認されたもの．

Ib期：臨床的に明らかな病巣が子宮頸部に限局するもの、または臨床的に明らかではないがIa期をこえるもの．

Ib1期：病巣が4cm以内のもの．

Ib2期：病巣が4cmをこえるもの．

II期：癌が子宮頸部を超えて広がるが骨盤壁または腟壁下1/3に達しないもの．

IIa期：腟壁に浸潤するが子宮傍組織へは浸潤しないもの．

IIb期：子宮傍組織に浸潤したもの．

III期：骨盤壁に浸潤したか腟壁下1/3に達したもの．

IIIa期：腟壁下1/3に達するが骨盤壁へは浸潤しないもの．

IIIb期：骨盤壁に浸潤したもの．

IV期：癌が骨盤腔を超えて広がるか，膀胱，直腸の結膜に浸潤したもの．

IVa期：膀胱，直腸の粘膜への浸潤があるもの．

IVb期：小骨盤腔を超えて広がるもの．

**3.2. 予後判定基準**

**3.2.1. 治療効果判定**

放射線治療終了１ヶ月後に治療効果判定を行う．

治療効果判定は，腫瘍縮小効果判定と細胞診により行う．

腫瘍縮小効果判定はMRI T2強調像にて行う．

標的病変が腫瘍による二次的変化も含めて消失した場合細胞診を行い，さらに必要に応じて組織

診を行う．

ここで，癌細胞の消失が確認された場合（Class 2以下と判定）のみ，治療効果判定をCRとし，それ

以外を全てnon-CRと判定する．

**3.2.2. 骨盤内増悪の定義**

骨盤内増悪とは，原発巣増悪と骨盤内リンパ節増悪の一方または両者を含むものである．増悪の判

定が困難な症例は，質的管理委員会にて検討する．

**原発巣増悪**

原発巣増悪の定義は以下の4通りとする．なお，原発巣増悪評価に関するベースラインは，放射線

治療終了1ヶ月後の治療効果判定結果とする．

1）治療効果判定にてCRと判定された例において，原発巣の再発を認めた場合．生検あるいは細胞

診による病理学的再発の確認を必要とする．

2）治療効果判定にてnon-CRと判定された例で，後に再増悪となった場合．再増悪とは治療効果判

定時点と比較してMRI T2強調像において長径が20％以上増大した場合，あるいは，視・触診に

て増悪が疑われた場合とする．

3）治療効果判定にてnon-CRと判定された例で，再増悪を確認されなかったが，原発巣に対する救

済治療（手術，化学療法等）が行われた場合．なお，この場合には救済治療が行われた日をもっ

てイベントとし，摘出物における癌細胞の残存の有無等は問わない．

4）治療効果判定が行われる前，すなわち標準治療経過中あるいは終了直後に救済治療が行われ

た場合には救済治療が行われた日をもってイベントとし，摘出物における癌細胞の残存の有無等

は問わない．

**骨盤内リンパ節増悪**

骨盤内リンパ節増悪とは，画像診断（CT，MRIあるいはFDG-PET/CT）にて骨盤内リンパ節の増大

を認めた場合をいう．骨盤内リンパ節増悪日は画像診断（CT，MRIあるいはFDG-PET/CT）の検査

日とする．生検による確認は必須としない．

**3.2.3. 遠隔転移発生の定義**

遠隔転移発生とは，画像診断あるいは視・触診等にて骨盤外の病変の新たな出現を認めた場合を

いう．骨盤外の病変とは，1)肺・肝臓・骨等の血行性転移，2)所属リンパ節以外のリンパ節転移（傍大

動脈リンパ節・鎖骨上リンパ節・鼠径リンパ節等），3)外陰部転移，4)癌性腹水等をさすものとする．

遠隔転移発生日は画像診断あるいは視・触診等で確認された日とする．生検による確認は必須とし

ない．腫瘍マーカーの上昇のみでは遠隔転移発生とはしない．

**3.2.4. 増悪の定義**

　　　骨盤内増悪または遠隔転移発生をもって増悪とする．

**4. 症例基準**

**4.1. 適格基準**

1. 原発巣が子宮頸部扁平上皮癌であることが組織学的に確認されている患者
2. FIGO臨床病期(1994年)がⅠb～Ⅳa期の患者
3. 一般状態(ECOG Performance Status, PS)が0～2の患者
4. 登録時の年齢が20歳以上85歳以下の患者
5. 傍大動脈リンパ節転移陰性の患者
6. 子宮頸癌に対して初回治療の患者
7. 本試験の参加について十分な説明を受けて趣旨を理解した上で文書に同意した患者

**4.2. 除外基準**

1. 断端癌患者
2. 活動性の重複癌を有する患者．ここでは，同時性重複癌及び無病期間が5年以内の異時性重複癌をいう．ただし，局所療法により治癒と判断されるCarcinoma in Situは活動性の重複癌には含めない．
3. 妊娠中，妊娠の可能性がある患者，もしくは授乳中である患者．
4. 挙児を希望している患者
5. 精神病または精神症状を合併しており試験への参加が困難な患者
6. 治療の実施に支障を来すと判断される重篤な合併症（膠原病・制御困難の糖尿病）を有する患者
7. 3ヶ月以内の心疾患の既往がある患者，もしくは慢性心不全の患者
8. 3ヶ月以内の重篤な脳血管障害の既往がある患者
9. 活動性の感染症を有する患者
10. 心臓ペースメーカーをしている患者
11. HBs抗原が陽性の患者
12. その他，臨床試験担当医師が本試験の対象として不適当と判断した患者

**4.3. 休止・再開基準**

本試験に登録された症例で以下の毒性を認めた場合は，回復するまで放射線治療は休止し，

回復後に再開する．

1） NCI-CTC (version 4.0日本語訳 JCOG版) によるGrade 4以上の血液毒性

2） NCI-CTC (version 4.0日本語訳 JCOG版) によるGrade 3以上の下痢，頻尿，悪心，放射線皮膚炎

3） PS 3

4） 合併基礎疾患の悪化のため治療の継続が困難とされた場合

5） 担当医が休止を必要と認めた場合

**4.4. 中止基準**

本試験に登録された症例で以下に示す条件が出現した場合，その症例におけるプロトコル治

療を中止する．

1) 治療開始後に原病の増悪が認められた場合

2) 有害事象によりプロトコル治療が継続できない場合

a. NCI-CTC (version 4.0日本語訳 JCOG版) によるGrade 4以上の下痢，頻尿，悪心，放射線皮膚炎

b. NCI-CTC (version 4.0日本語訳 JCOG版) によるGrade 4の血液毒性による治療休止が21日以上遷延した場合

c. NCI-CTC (version 4.0日本語訳 JCOG版) によるGrade 3の下痢，頻尿，悪心，放射線皮膚炎による治療休止が21日以上遷延した場合

3) 担当医が，毒性により放射線治療が継続不可能と判断した場合

4) PS4

5) 合併基礎疾患の悪化による治療休止が21日以上遷延した場合

6) 患者が治療の継続を拒否した場合

**4.5. 脱落基準**

パイロット研究（子宮頸癌の放射線治療予後予測因子としてのアポリポ蛋白C-II，第48回日本癌治療学会2010，播磨ら）における再発イベント発生時期（2.3～34.1ヶ月）の第3四分位が12.2ヶ月であるところから，最短観察期間を12ヶ月とした．したがって，登録後，治療完遂を目途として経過観察を実施するが，最短観察期間12ヶ月未満の打ち切り例については，観察期間不足症例として解析から除外する．

**5. 登録**

　　　データセンターを設定し，中央登録方式を採用する．

担当医は対象患者が適格基準を全て満たし，かつ除外基準のいずれにも該当しないことを確認し

た後，登録票に必要事項をすべて記入の上，データセンターにFAX送信する．

データセンター

近土写真製版株式会社メディカル事業部

連絡先：〒543-0011大阪市天王寺区清水谷町11-15

TEL：0120-619-198

ＦＡＸ：0120-219-199

メールアドレス：ito@mydo-kond.co.jp

時間：平日9時から17時まで（土日，祝祭日を除く）

■問い合わせ先

適格基準など：関西医科大学滝井病院放射線科　准教授　播磨洋子

登録手続き：近土写真製版株式会社メディカル事業部　伊東俊幸

**6. 実施方法**

バイオマーカーApoC-IIの測定

①血液採取とMMP1，MMP2，SCCの測定

放射線治療前と放射線治療終了1ヶ月後に6mLの採血を行い，ベノジェクトⅡ真空採血管に速やかに検体を注入し室温に30～60分静置させ，凝固を確認後に室温で保管する．全血を各施設の検査室で3000rpm10～15分間遠心分離後，上清を提出容器に移し，株式会社エスアールエル（SRL）が回収し凍結保存する。

SRLはMMP1，MMP2，SCCを測定する．測定後検体を関西医科大学放射線科に移送し保管する．検査結果はSRLから研究事務局に封書で送られる．封書は開封されずデータセンターに送付される．

②ApoC-IIモノクロナール抗体Elisa-Kitの測定

関西医科大学放射線科から株式会社医学生物学研究所（MBL）に検体を移送する．MBLは関西医科大学放射線科が作成したApoC-IIモノクロナール抗体Elisa-Kitに集積した患者血清を反応させ，その値を測定する．

検査結果はMBLから研究事務局に封書で送られる．封書は開封されずデータセンターに送付される．

**7. スタディカレンダー（検査日程）**

①登録時に次の項目について記録する．これらは登録前2週間までのものを許容する．

年齢（生年月日），FIGO臨床病期(1994年)，一般状態(ECOG PS)，身長，体重，腫瘍最大径，骨盤内リンパ節転移有無，血液検査（血球・生化学）

また，画像診断（CT，MRIあるいはFDG-PET/CT）による確認を必須とする．

　　　②登録後，治療開始が何らかの理由で遅延した場合（白血球低下など）は，治療開始基準を満たす

まで待つ．登録から治療開始までの期限は特に定めない．

③治療終了時に次の項目について記録する．

放射線治療開始日・終了日，骨盤照射（中央遮蔽なし）線量，骨盤照射（中央遮蔽あり）線量，腔

内照射1回線量，腔内照射総線量，化学療法（なし，あり），化学療法薬剤名・用量・コース数

④登録時と放射線治療終了1ヶ月後に，6mLの静脈血を採取し，下記項目の測定に使う．

ApoC-II，MMP1，MMP2, SCC

ApoC-II，MMP1，MMP2の測定結果については治療者にマスクされる．

⑤放射線治療終了１ヶ月後に治療効果判定を行う．

⑥以下の項目については，治療終了3ヶ月後，6ヶ月後，1年後，2年後に経過観察を実施する．

観察項目：転帰（生存，増悪，死亡）とイベント発生日

⑦再発が疑われた場合には内診，画像診断（CTなど）を適宜行う．局所再発の確認には病理診断

が望ましい．

|  | 放射線治療前（登録時） | 放射線治療 | 放射線治療終了１ヶ月後 | 放射線治療終了３ヶ月後 | 放射線治療終了６ヶ月後 | 放射線治療終了12ヶ月後 | 放射線治療終了24ヶ月後 | 中止・終了時 |
| --- | --- | --- | --- | --- | --- | --- | --- | --- |
| 日付（検査日・診察日） | ● |  | ● | ● | ● | ● | ● | ● |
| 生年月日 | ● |  |  |  |  |  |  |  |
| ECOG Performance Status | ● |  |  |  |  |  |  |  |
| 身長・体重 | ● |  |  |  |  |  |  |  |
| FIGO臨床進行期分類 | ● |  |  |  |  |  |  |  |
| 腫瘍最大径 | ● |  |  |  |  |  |  |  |
| リンパ節転移の有無 | ● |  |  |  |  |  |  |  |
| 治療内容（放射線ほか治療内容） |  |  | ● |  |  |  |  |  |
| 血液検査（血球・生化学） | ● |  | ● |  |  |  |  |  |
| SCC | ● |  | ● |  |  |  |  |  |
| MMP1・MMP2 | ● |  | ● |  |  |  |  |  |
| ApoC-II | ● |  | ● |  |  |  |  |  |
| 治療効果判定 |  |  | ● |  |  |  |  |  |
| 内診 |  |  | ● | ● | ● | ● | ● | ● |
| CT（胸部・腹部・骨盤部） |  |  |  |  | ● | ● | ● |  |
| 転帰（最終確認日における患者の状態） |  |  |  | ● | ● | ● | ● | ● |

●各医療施設において測定・記録．●下記の特定検査機関にて測定

・ApoC-IIは株式会社医学生物学研究所（MBL）にて測定．

・MMP1, MMP2, SCCは株式会社エスアールエル（SRL）にて測定．

・血液検査として赤血球，白血球，血小板，Hb，総蛋白，Alb，AST(GOT)，ALT(GPT)，BUN，Cr，eGFR，

CRPを測定する．

・通常の血液検査（血球・生化学）のための採血と，SCC・MMP1・MMP2・ApoC-II測定のための採血は同時

に行われるのが理想的であるが，放射線治療前については，すでに他科で通常の血液検査を済ませていること

が多いかも知れない．その場合はSCC・MMP1・MMP2・ApoC-II測定のための採血から遡って2週間以内

の他科における通常の血液検査結果を許容するものとする．登録時に必要なその他の検査についても同様に2

週間以内のものを許容する．

**8. データ収集**

**8.1. 記録用紙の種類と提出期限**

症例記録用紙（CRF）への必要データの記載は各研究協力施設の施設研究者もしくはデータ管理者がもれなく記載しなければならない．症例記録用紙原本は下記の時期にデータセンターが回収する．

| 用紙の名称 | データセンターの回収時期 |
| --- | --- |
| 登録票（登録適格性確認票）  調査票該当頁（登録時データ・治療内容・血液学的  検査） | 登録時（FAX送付） |
| 調査票該当頁（血液学的検査・治療効果判定） | 放射線治療終了1ヶ月後（FAX送付） |
| 調査票該当頁（イベント発生報告） | イベント（骨盤内増悪・遠隔転移・死亡など）発生時（FAX送付） |
| 調査票該当頁（有害事象・転帰など） | 中止・脱落時，観察終了時（FAX送付） |

**8.2. 調査用紙の送付方法**

登録票および調査票の該当頁を所定の時期にデータセンターにFAXする．

**9. 有害事象**

**9.1. 有害事象の評価**

有害事象共通用語基準v4.0日本語訳JCOG版を用いて有害事象を評価し，記録用紙（CRF）に記録する．

**9.2. 予想される有害事象**

**CDDPによるもの**

急性腎不全，汎血球減少症，ショック／アナフィラキシー様症状，聴力低下，難聴，耳鳴り，鬱血乳

頭，球後視神経炎，皮質盲，脳梗塞，血栓性微小血管症，心筋梗塞，鬱血性心不全，溶血性貧血，間質性肺炎

**放射線治療によるもの**

1）早期（放射線治療開始日より90日以内）

倦怠感，悪心・嘔吐，下痢，頻便，頻尿，皮膚発赤，陰部脱毛，血球減少

2）晩期（放射線治療開始日より91日以降）

直腸炎，直腸潰瘍，軟便，小腸炎，頻尿，血尿，膀胱膣ろう，直腸膣ろう，骨折，下肢浮腫，蜂

窩織炎

**9.3.　有害事象発生時の対応**

　　　本研究では標準的な治療が実施されるので，CRFへの記録以外は特に規定せず，各施設の規定

に基づいて対応する．

**10. 効果判定とエンドポイントの定義**

**10.1.　効果判定**

　　放射線治療終了１ヶ月後に治療効果判定を行う．

治療効果判定は，腫瘍縮小効果判定と細胞診により行う．

腫瘍縮小効果判定はMRI T2強調像にて行う．

標的病変が腫瘍による二次的変化も含めて消失した場合細胞診を行い、さらに必要に応じて

組織診を行う．

ここで，癌細胞の消失が確認された場合（Class 2以下と判定）のみ，治療効果判定をCRと

し，それ以外を全てnon-CRと判定する．

**10.2. エンドポイント**

**10.2.1. プライマリーエンドポイント**

無増悪生存期間：

放射線治療開始日をもって観察開始日とし，骨盤内増悪および遠隔転移発生またはあらゆる原

因による死亡をイベントとする．

病理診断やFDG-PET/CTなどで増悪が確認された日を増悪確定日とする．

イベント非発生例では最終確認日をもって打ち切りとする．

**10.2.2. セカンダリーエンドポイント**

全生存期間：

放射線治療開始日をもって観察開始日とし，あらゆる原因による死亡をイベントとする．

生存例では最終生存確認日をもって打ち切りとする．追跡不能例では追跡不能となる以前で生

存が確認されていた最終日をもって打ち切りとする．

骨盤内無増悪生存期間：

放射線治療開始日をもって観察開始日とし，骨盤内増悪をイベントとする．

病理診断やFDG-PET/CTなどで骨盤内増悪が確認された日を骨盤内増悪確定日とする．

骨盤内増悪が無い状態で，遠隔転移を含む他の原因にて死亡した場合には，死亡日をもって打

ち切りとする．

遠隔転移無発生生存期間：

放射線治療開始日をもって観察開始日とし，遠隔転移発生をイベントとする．

病理診断やFDG-PET/CTなどで遠隔転移発生が確認された日を遠隔転移発生確定日とする．

生検による確認は必須としない．腫瘍マーカーの上昇のみでは遠隔転移発生としない．

遠隔転移が無い状態で，骨盤内増悪を含む他の原因にて死亡した場合には，死亡日をもって打

ち切りとする．

**11. 統計的事項**

**11.1. 中間解析と試験の早期中止**

中間解析は実施しない．

**11.2. 最終解析**

**前向きコホート研究**

1)プライマリーエンドポイントの解析

Kaplan-Meier法による生存率曲線およびその95％信頼区間を推定する．

イベント発生までの期間を従属変数としたCox比例ハザードモデルを構築する．

ApoC-II Elisa-Kit発現量別イベント発生までの期間の比較にはLog-Rank検定を行う．

2)セカンダリーエンドポイントの解析

プライマリーエンドポイントの解析結果を補足する目的としてKaplan-Meier法による生存率曲線およびその95％信頼区間を推定する．

イベント発生までの期間を従属変数としたCox比例ハザードモデルを構築する．

ApoC-II Elisa-Kit発現量別イベント発生までの期間の比較にはLog-Rank検定を行う．

3)相関分析

ApoC-II Elisa-Kit発現量とMMP１，MMP2，の相関分析を行う．変数の分布の正規性が認められた場合にはPearsonの相関係数を，正規性が認められなかった場合にはSpearmanの相関係数を採用する．

4)各マーカーの性能評価

ApoC-IIおよびMMP1，MMP2，SCCについてプライマリーエンドポイント及びセカンダリーエンドポイントを推定する性能評価についてROC解析を実施する．

その上でそれぞれのマーカーのカットオフポイントを同定し感度，特異度を算出する．

ROC解析についてはAUC(Area Under Curve)面積を算出しHanley^22)^の方法（AUCの差を差の標準誤差で除すことで得られるZ値を評価する）による比較検定を実施する．

カットオフポイントは連続的に推移する感度－（１－特異度）の最も大きな値が得られたマーカー値とする．

その他，マーカーとしての性能を評価するために，本試験で得られた測定値をもとに探索的な解析を実施する．

**ネステッド・ケース・コントロール研究**

　　　1)プライマリーエンドポイントおよびセカンダリーエンドポイントの解析

　　　　 ApoC-II等の曝露要因についてt検定を行う．

イベントの発生・非発生を従属変数としたロジスティック回帰モデルを構築する．独立変数は関心

の対象としてはApoC-IIを，調整因子としてはFIGO臨床病期，SCC，BMI，化学療法の有無を投入する．

**12.　倫理的事項**

**12.1.　患者の保護**

本試験に関係するすべての研究者はヘルシンキ宣言に従って本試験を実施する．

また，本試験は「臨床研究に関する倫理指針（厚生労働省）」に従って実施される．

**12.2.　インフォームド・コンセント**

インフォームド・コンセントの取得無しに本研究に登録してはならない．

**12.2.1.　患者への説明**

登録に先立って，担当医は患者本人に別紙の開示文書もしくは施設の機関審査委員会の承認が

得られた説明文書を渡し，以下の内容を口頭で詳しく説明する．

1）病名，病期，推測される予後に関する説明

2）本試験が臨床試験であること

3）本試験のデザインおよび根拠（意義，登録数，必要性，目的など）

4）プロトコール治療の内容

資料採取のための定期的な採血以外は標準的な治療であること．

5）費用負担と補償

治療にかかる費用は保険制度でまかなわれること，健康被害が生じた場合の補償は一般診療での対処に準ずることなど，一般診療と同様であることを説明する．生化学検査として赤血球，白血球，血小板，Hb，総蛋白，Alb，AST(GOT)，ALT(GPT)，BUN，Cr，eGFR，CRP, SCCを測定し，資金は保険診療でまかなわれる．MMP1, MMP2，SCCは株式会社エスアールエル（SRL）にて測定し，ApoC-IIは株式会社医学生物学研究所（MBL）にて測定する．それぞれの資金は本研究をテーマにした平成22年度～平成24年度基盤研究（Ｂ）一般 (課題番号22390237)「分子生物学的手法を用いた進行期子宮頸癌の放射線治療抵抗性機構の解明とその克服」（1620万円，研究代表者：播磨洋子，連携研究者：JROSG婦人科腫瘍グループ）を用いる．

6）試験に参加することで患者に予想される利益と可能性のある不利益

資料採取のために，通常の診療に伴う採血量が12 mLほど増加するが，これによる侵襲の増加は

軽微であり，副作用等の生じる恐れはほとんどない．被験者に万一採血による事故や後遺症が生

じた場合，施設の医療事故対策ガイドラインに準じて適切かつ迅速に対処し，保険診療内におけ

る最善の治療を行う．ただし，通常の保険診療と同様特別な補償はないが，万一賠償問題が発生

すれば通常の医療事故と同様に誠実に対応する．

7）同意拒否と同意撤回

試験参加に先立っての同意拒否が自由であることや，一旦同意した後の同意の撤回も自由であり，それにより不当な診療上の不利益を受けないこと．また，研究終了後でも同意の撤回ができる権利があり，研究終了後に同意撤回があった場合は，資料・データを返還もしくは破棄することを説明する．

8）人権保護

個人情報が研究から発表に至る全過程で一切公開されないよう厳重管理するために，各施設の

研究責任者が以下の管理方法を用いる．すなわち，①検体提供者の氏名・性別・生年月日を削

除し，記号を不作為にふることにより匿名化し，研究責任者を除いては被験者個人を特定できな

くする．② 研究責任者が，対象者の情報を決して他人には知らせない．③ 研究責任者だけが

使用可能なオフラインのコンピューターで厳重に保管し（連結可能匿名化），全資料（個人情報

の匿名化過程を含む）を保管する．

研究成果は集団としての結果を学会，学術雑誌に公表するが，個人を特定できる情報としては

一切公表しない．

9)データの二次利用

　　　　個人識別情報とリンクしない形でデータをメタアナリシスなどに二次利用する可能性があることを

説明する．

　　　10)検体の扱い

　　　　　研究終了後早急に破棄することを説明する．

11）質問の自由

担当医の連絡先のみでなく，施設の研究責任者，試験の研究代表者（または研究事務局）の連

絡先を文書で知らせ，試験や治療内容について自由に質問できることを説明する．

**12.2.2.　同意の取得**

説明を行った翌日以降，患者が臨床試験の内容をよく理解したことを確認した上で，試験への 参加について依頼する．患者本人が試験参加に同意した場合は，説明，同意文書末尾に自署 による署名を得る．同意書原本は説明した医師の署名捺印，同意を得た日付を記載した文書と して診療録（カルテ）に保存する．同意書コピー1部を作成し，説明文書と共に患者に手渡しをする．

**12.3.　プライバシーの保護と患者識別**

登録患者の氏名は参加施設からデータセンターへ知らされることはない．登録患者の同定や照会は，登録時に発行される登録番号とカルテ番号を用いて行われる（連結可能匿名化）．患者名など直接患者を識別できる情報がデータセンターのデータベースに登録されることはない．施設，データセンター，研究事務局間の患者データのやりとりは，紙，電子媒体のいかんにかかわらず，症例登録と緊急性を要する有害事象報告を除き，郵送あるいは直接手渡しすることを原則とする．

**12.4.　プロトコールの遵守**

本試験に参加する研究者は，患者の安全と人権を損なわない限りにおいて本研究実施計画書を遵

守する．

**13．研究費用および利益相反**

**13.1．研究費用**

　　　本研究をテーマにした平成22年度～平成24年度基盤研究（Ｂ）一般 (課題番号22390237)「分子

生物学的手法を用いた進行期子宮頸癌の放射線治療抵抗性機構の解明とその克服」（1620万円，

研究代表者：播磨洋子，連携研究者：JROSG婦人科腫瘍グループ）を用いる．

**13.2．利益相反**

　　　本研究に関して開示すべき利益相反はない．

**14. モニタリングと監査**

**14.1. 定期モニタリング**

試験が安全に行われているか，データが正確に収集されているか，試験がプロトコールの規定どおりに実施されているかを確認する目的で，原則として年1回定期モニタリングが行われる．モニタリングはデータセンターに収集される記録用紙（CRF）の記入データに基づいて行われる中央モニタリングであり，施設訪問にて原資料との照合を含めて行う施設訪問モニタリングは予定していない．

定期モニタリングの結果は文書としてまとめられ，各研究連携者に報告される．

**14.1.1. 定期モニタリングの項目**

1）症例集積達成状況：登録症例数，累積，期間別

2）適格性：不適格例

3）放射線治療の適正性

4）有害事象の集計

5）プロトコール逸脱

6）生存に関する記載

**14.2. 施設訪問監査**

必要に応じて放射線治療内容について施設訪問監査を行い，放射線治療精度を検証する．

**15.　研究組織**

**15.1.　研究代表者**

関西医科大学　滝井病院　放射線科　准教授　播磨洋子

連絡先：〒570-8507　大阪府守口市文園町10-15

TEL: 06-6992-1001

FAX: 06-6993-3865

E-mail: harima@takii.kmu.ac.jp

**15.2.　参加施設および研究連携者**

NPO-JROSG婦入科腫瘍グループは以下の研究施設，研究連携者(職位)である．（順不同）

1. 群馬大学大学院医学系研究科放射線科　中野　隆史（教授）
2. 埼玉医科大学総合医療センター放射線腫瘍科　高橋　健夫（教授）
3. 群馬大学重粒子線医学研究センター放射線科　大野　達也（教授）
4. 琉球大学放射線科　戸板　孝文（准教授）
5. 徳島大学大学院ヘルスバイオサイエンス研究部医用情報科学講座　生島　仁史（教授）
6. 静岡県立静岡がんセンター放射線治療科　西村　哲夫（部長）
7. 埼玉医科大学国際医療センター放射線腫瘍科　加藤　眞吾（教授）
8. 広島大学大学院医歯薬学総合研究科放射線治療科　兼安　祐子（助教）
9. 北里大学病院放射線腫瘍学科　新部　譲（講師）
10. 埼玉県立がんセンター放射線科　楮本　智子（副部長）
11. 佐賀大学医学部重粒子がん治療学講座　徳丸　直郎（教授）
12. 聖マリアンナ医科大学放射線科　五味　弘道（講師）
13. 関西医科大学滝井病院放射線科　播磨洋子（准教授）

**15.3.　データセンター**

近土写真製版株式会社メディカル事業部

責任者：伊東俊幸

連絡先：〒543-0011大阪市天王寺区清水谷町11-15

TEL：0120-619-198

FAX：0120-219-199

E-mail:ito@mydo-kond.co.jp

時間：平日9時から17時まで（土日，祝祭日を除く）

■問い合わせ先

●適格基準など：関西医科大学附属滝井病院放射線科

責任者：播磨洋子

連絡先：〒570-8507守口市文園町10-15

TEL：06-6992-1001

FAX：06-6993-3865

E-mail：harima@takii.kmu.ac.jp

●登録手続き：近土写真製版株式会社メディカル事業部

責任者：伊東俊幸

**15.4. プロトコール作成責任者**

関西医科大学　滝井病院　放射線科　准教授　播磨洋子

連絡先：〒570-8507　大阪府守口市文園町10-15

TEL: 06-6992-1001

FAX: 06-6993-3865

E-mail: harima@takii.kmu.ac.jp

**15.5.　質的管理委員会**

委員長：播磨洋子 関西医科大学　滝井病院 放射線科

委員：中野隆史 群馬大学大学院医学系研究科放射線科

委員：高橋健夫　　　 埼玉医科大学総合医療センター放射線腫瘍科

委員：大野達也 群馬大学重粒子線医学研究センター放射線科

委員：戸板孝文 琉球大学放射線科

委員：生島仁史 徳島大学大学院ヘルスバイオサイエンス研究部医用情報科学講座

委員：西村哲夫 静岡県立静岡がんセンター放射線治療科

委員：加藤眞吾 埼玉医科大学国際医療センター放射線腫瘍科

委員：兼安祐子 広島大学大学院医歯薬学総合研究科放射線治療科

委員：新部譲 北里大学病院放射線腫瘍学科

委員：楮本智子 埼玉県立がんセンター放射線科

委員：徳丸直郎 佐賀大学医学部重粒子がん治療学講座

委員：五味弘道 聖マリアンナ医科大学放射線科

**16.　研究成果の発表**

主たる公表論文，学会発表は，最終解析終了後に質的管理委員会の承認を経て，専門誌（英文

誌）に投稿し，あるいは専門学会に発表する．

【論文発表に関するauthorship等に関する取り決め】

原則として論文発表に関するauthorshipは以下の通りとする．

試験結果の公表論文の1st authorは研究代表者とする．2nd authorは症例登録数が最も多かった

施設の実務担当者もしくは代表者（該当施設内の筆頭者の選択は，その施設の判断による．ただし

研究に最も貢献した者を原則とする）とする．ただしそのauthorshipを取得するかどうかは当該施設

の選択による．それ以外は，論文の投稿規程による制限に従って，登録数の多い順に選び共著者と

する．

すべての共著者は投稿前に論文内容をreviewし，発表内容に合意した者のみとする．内容に関し

て議論しても合意が得られない場合，研究代表者は質的管理委員会の了承の上で，その研究者を

共著者に含めないことができる．

学会発表は複数回に及ぶ可能性があり，1.研究代表者，2.症例登録数が最も多かった施設の代表

者，3.登録数が多い順の施設代表者の優先順位で発表する権利を与える．

**17.　参考文献**

1）Franco E et al. Human Papillomavirus DNA in Invasive Cervical Carcinomas and Its Assciation with Patient Survival: A Nested Case-Control Study. Cancer Epidemiol Biomarkers Prev. 1996 Apr;5(4):271-5.

2）長谷川久美他　閉塞性大腸癌の検討　日消外会誌 33 (6):709-15, 2000年

3）播磨洋子　進行子宮頸癌の放射線治療予後に関与するアポリポ蛋白C-II　第69回日本癌学会学術総会抄録 p.463, 2010

4) Kim SY et al. Apolipoprotein C-II is a novel substrate for matrix metalloproteinases. Biochem Biophys Res Com 339: 47-54, 2006

5) Zhai Y et al. Expression of Membrane Type 1 Matrix Metalloproteinase Is Associated with Cervical Carcinoma Progression and Invasion. Cancer Res 65: 6543-6550, 2005

6) Harima Y et al. Identification of genes associated with progression and metastasis of advanced cervical cancers after radiotherapy by cDNA microarray analysis. Int J Radiat Oncol Biol Phys 75:1232-1239, 2009

7) 子宮頸癌治療ガイドライン2011年度版　日本婦人科腫瘍学会編　金原出版株式会社

8) Cohen, J. Statistical power analysis for the behavioral sciences (2nd ed.). Hillsdale, NJ: Lawrence Earlbaum

Associates, 1988

9) Noordhuis MG et al. Prognostic cell biological markers in cervical cancer patients. Primarily treated with (chemo)radiation: a systematic review. Int J Radiat Oncol Biol Phys 79: 325–334, 2011

10) Kim GE et al. Synchronous coexpression of epidermal growth factor receptor and cyclooxygenase-2 in carcinomas of the uterine cervix: A potential predictor of poor survival. Clin Cancer Res 10:1366–1374, 2004

11) Perez-Regadera J et al. Negative prognostic impact of the coexpression of epidermal growth factor receptor and c-erbB-2 in locally advanced cervical cancer. Oncology 76:133–141, 2009

12) Kim JY et al. Tumor-associated carbonic anhydrases are linked to metastases in primary cervical cancer. J Cancer Res Clin Oncol 132: 302–308, 2006

13) Vaupel P et al. Hypoxia in cancer: Significance and impact on clinical outcome. Cancer Metastasis Rev 26:225–239, 2007

14) Ogino I et al. The role of pretreatment squamous cell carcinoma antigen level in locally advanced squamous cell carcinoma of the uterine cervix treated by radiotherapy. Int J Gynecol Cancer 16:1094–1100, 2006

15) [Gadducci A](http://www.ncbi.nlm.nih.gov/pubmed?term=%22Gadducci%20A%22%5BAuthor%5D) et al. The serum assay of tumour markers in the prognostic evaluation, treatment monitoring and follow-up of patients with cervical cancer: a review of the literature. Crit Rev Oncol Hematol 66:10-20, 2008

16) Chen J et al. Characterization of apolipoprotein and apolipoprotein precursors in pancreatic cancer serum samples via two-dimensional liquid chromatography and mass spectrometry. J Chromatogr A 1162:117-125, 2007

17) de Wit NJ et al. Differentially expressed genes identified in human melanoma cell lines with different metastatic behaviour using high density oligonucleotide arrays. Melanoma Res 12:57-69, 2002

18) Chum EM, Expression of the apolipoprotein C-II gene during myelomonocytic differentiation of human leukemic cells. J Leukoc Biol. 69:645-50. 2001

19) Murray GI et al.[Matrix metalloproteinase-1 is associated with poor prognosis in colorectal cancer.](http://www.ncbi.nlm.nih.gov/pubmed/8597958) Nat Med. 2:461-462, 1996

20) Inoue T et al. [Matrix metalloproteinase-1 expression is a prognostic factor for patients with advanced gastric cancer.](http://www.ncbi.nlm.nih.gov/pubmed/10373641) Int J Mol Med. 4: 73-77, 1999

21) Sheu BC et al. Increased Expression and Activation of Gelatinolytic Matrix Metalloproteinases Is Associated with the Progression and Recurrence of Human Cervical Cancer. Cancer Res 63:6537-6542, 2003

22) Hanley, JA, McNeil BJ. A method of comparing the areas under receiver operating characteristic curves derived from the same cases. Radiology 148:839-43, 1983

検査機関

　　　株式会社エスアールエル　（SRL）

　　　東京都立川市曙町2-41-19損保ジャパン立川ビル9F

TEL：042-526-7111（代）

株式会社医学生物学研究所　（MBL）

名古屋市中区丸の内3-5-10住友商事丸の内ビル5F
TEL:052-971-2081（代）

**18.　付表Appendix**

1）説明文書・同意書

2）ヘルシンキ宣言（日本医師会和訳）

3）ECOG のPerformance Status（PS）の日本語訳

4）記録用紙（CRF）

・登録票

・登録確認票

・調査票

5）有害事象共通用語基準 v4.0日本語訳JCOG版
