# Supplementary material for: Clinical value of serum biomarkers, squamous cell carcinoma antigen and apolipoprotein C-II in follow-up of patients with locally advanced cervical squamous cell carcinoma treated with radiation: A multicenter prospective cohort study
Source: PLoS One. 2021 Nov 2;16(11):e0259235. doi: 10.1371/journal.pone.0259235 (PMC8562853; doi:10.1371/journal.pone.0259235)
Supplement: S3 File — (DOCX) [file pone.0259235.s004.docx]

**The main points of clinical study protocol** (Translation from Japanese to English)

**Title of Study**

Clinical value of serum biomarkers, squamous cell carcinoma antigen and apolipoprotein C-II in follow-up of patients with locally advanced cervical squamous cell carcinoma treated with radiation: A multicenter prospective cohort study

**Research contact person**: Yoko Harima

**Organization**: Kansai Medical University

**Division**: Radiology

**Address**: 10-15, Fumizono-cho, Moriguchi, Osaka, Japan

Tel: +81-6-6992-1001

Email: harima@takii.kmu.ac.jp

**Research Institute**: Kansai Medical University

**Ethical Review Committee**: The ethical committee at Kansai Medical University

**Approved Date**: 25, January 2012.

**Reception No. 438**

**Approval No. 0772**

**Financial support**: This study was supported by grants B 22390237 and B 25293266 from a Grant-in-Aid for Scientific Research from the Ministry of Education, Science, Sports and Culture, Japan. This study was also supported by grants JROSG 10-4 from the Japanese Radiation Oncology Study Group.

**Trial Registration umin.ac.jp/ctr Identifier**: 000004467

**Conflict of interest**: None.

**Synopsis**

**Study Protocol and Ethical Consideration**

| Study objective | To identify serum biomarkers for survival after radiotherapy for cervical cancer. |
| --- | --- |
| Study Design | A multicenter prospective cohort study. |
| Participants | 1. Patient with histologically proven squamous cell carcinoma of the uterine cervix; International Federation of Gynecology and Obstetrics (FIGO) stage IB–IVA.  2. Patient with Eastern Cooperative Oncology Group (ECOG) performance status, 0–2.  3. Patient aged 20–85 years.  4. Patient with no para-aortic lymph node metastasis  5. Patient with no history of radiotherapy, chemotherapy, or surgery for cervical cancer  6. Patient who joined in the explanation session and gave written informed consent. |
| Target sample size | 150 |
| Exclusion criteria | 1. Patient with cervical stump carcinoma.  2. Patient with active synchronous or metachronous (< 5 years) double cancers.  3. Patient who is pregnant or breastfeeding.  4. Patient trying to conceive.  5. Patient with uncontrolled concurrent medical or neurological conditions.  6. Patient with serious complications affecting treatment, including connective tissue disorders; uncontrolled diabetes; serious, chronic heart failure or cerebrovascular disorder in the 3 months prior to study enrollment.  7. Patient with active infection.  8. Patient using pacemaker.  9. Ineligibility based on the investigator’s assessment. |
| Groups | N/A |
| Intervention | Measurement of serum biomarkers including apolipoprotein C-II, squamous cell carcinoma antigen, and matrix metalloproteinase1 and 2 before and 1 month after radiotherapy |
| Primary outcomes | Progression-free survival (PFS) |
| Secondary outcomes | Overall survival (OS), pelvic PFS (PPFS), and distant metastasis-free survival (DMFS). |
| Approval of ethical committee and informed consent | The study protocol was approved by the Kansai Medical University Review Board. Before participating the study, the potential participants must join in study information session with sufficient detail of the study including the purpose, the method and ethical consideration and signed the informed consent form approved by the institutional review board of each center.  Further, the participants are guaranteed the right to withdraw their consent after entering the study. |
| Data Management | Data management was performed by a contracted research organization (Kondo P.P.Inc). The anonymized data with identification number excluding names, addresses or telephone numbers are stored in the locked cabinet of each center for five years after this study finished. |
| Consultation from participants | If the participants hope to ask any questions or withdraw their consent, they can contact the investigators by referring the telephone number, e-mail address in the handout. |
| Publication | The findings of this study will be published in medical journal or presented at scientific meetings. |
| Financial Disclosure | This study was supported by grants B 22390237 and B 25293266 from a Grant-in-Aid for Scientific Research from the Ministry of Education, Science, Sports and Culture, Japan. This study was also supported by grants JROSG 10-4 from the Japanese Radiation Oncology Study Group. |
| Conflict of Interest | The authors declare that no conflict of interests exists. |
| Planned follow-up period | 2012 March – 2015 February. |
